# Characterization of landslide dams in a sector of the central-northern Apennines (Central Italy)

**DOI:** 10.1016/j.heliyon.2020.e03799

**Published:** 2020-06-03

**Authors:** Corrado Cencetti, Pierluigi De Rosa, Andrea Fredduzzi

**Affiliations:** Department of Physics and Geology, University of Perugia - Via Pascoli s.n.c., 06125 Perugia, Italy

**Keywords:** Earth sciences, Geography, Geology, Natural hazards, Landslide database, Landslide dam hazard, River system

## Abstract

The paper analyses the landslide dam phenomenon in an area of the central-northern Apennines (Italy). A census of 53 landslides was carried out, identifying the salient parameters of both the landslides and the riverbed–alluvial plain systems. Correlations between these parameters were tested, in order to characterize the phenomenon in the study area. The most interesting relationships are those that compare the volume of landslide material either with the area of the catchment subtended by the dammed section and with the width of the riverbed–alluvial plain system. The results indicate that as the width of the fluvial system increases, and the area of the river basin subtended by the natural dam increases, the volume of the landslide accumulation necessary to produce occlusion also increases. Only one case, among those examined, escapes this general rule: a landslide of small volume made a full occlusion even though a relatively large area of basin was subtended. In this specific case, landslide velocity played an important role. So, while a high ratio between the volume of the landslide and the area of the affected basin represents a predisposing condition for the occurrence of total occlusion, it is not wholly reliable. Indexes, proposed in the literature, suggesting a quantitative assessment of landslide dam hazard, fail to forecast total occlusion unequivocally. This is because of the special characteristics of each territorial area, from lithological, geomorphological and geological-structural point of views. Such indexes, when they do not lose their intrinsic reliability (as in this case), must at least be identified with, and applied to, specific and restricted territories: they cannot be generalized.

## 1 Introduction

The interactions between riverbeds dynamics and gravitational slope phenomena are, in general, complex and not always easily predicted and defined. They range from a simple increase in debris supply from the slope, with a consequent increase in solid transport in the riverbed, to a partial or total occlusion of the riverbed by landslides, which can result in, under conducive conditions, the damming of an entire valley, with the formation of a lake (landslide dam).

The landslide dam phenomenon creates risks much greater than those arising from the gravitational process alone. Extreme cases (total damming of the riverbed - floodplain system) can lead to submergence of areas located upstream of the landslide dam, with the consequent possibility of overflowing and/or collapse of the dam itself, and hence rapid emptying of the dam lake and formation of an anomalous downstream wave (Costa, 1988; Costa and Schuster, 1988; Schuster, 1986, 2003; Tabata et al., 2001; Ermini and Casagli, 2003; Dunning et al., 2005; Evans, 2006; Chen et al., 2017; Fan et al., 2017; Diprizio et al., 2018; Paliaga et al., 2018).

Assessment of the risk connected to these events, and to vulnerable environments and populations, cannot be effectively evaluated if the two main components (hydraulic risk and landslide risk) are considered separately. On the one hand, the hydraulic hazard connected to riverbed occlusion by a landslide cannot be assessed in terms of “return times”, because it relates to an occasional phenomenon. On the other hand, landslides that in themselves can be considered to have low risk potential (because they involve a relatively small volumes of material, or because they do not directly impact sensitive structures or infrastructures) can cause high risk conditions through induced hydraulic phenomena on the riverbed–alluvial plain system; especially, occlusion and its possible subsequent evolution (submersion upstream of the natural dam and an anomalous wave due to a possible dam break).

Previous scientific papers on this topic focused on assessment of the landslide dam formation and dam break hazard. This has involved definition of pertinent indexes on the basis of specific inventories of landslides of this type (Schuster, 1986; Swanson et al., 1986; Pirocchi, 1991; Canuti et al., 1998; Casagli and Ermini, 1999; Ermini and Casagli, 2002, 2003; Ermini et al., 2006; Dong et al., 2011, 2014; Peng and Zhang, 2012; Qiao et al., 2013; Dal Sasso et al., 2014; Cencetti et al., 2015a; Ermini, 2018). More recently, Tacconi Stefanelli at al. (2015) produced a census of the most important known landslide dams in Italy (300 cases), and also introduced an index for the assessment of hazard resulting from riverbed occlusion by landslide (Tacconi Stefanelli et al., 2016).

This paper reports the results of a census of landslides that have historically interfered with riverbeds, causing partial or total occlusion, in a particular sector of the central-northern Apennines (central Italy), with the aim of understanding the modalities which characterise past occurrences. The area investigated (27,230 km^2^) includes the whole regions of Umbria and Marche and their borders with neighbouring regions (Latium, Abruzzo, Emilia-Romagna, Tuscany).

The detail with which the survey was conducted was significantly greater than in previous works, such that 53 cases were identified. Tacconi Stefanelli et al. (2015) found just 25 cases in the same area.

The results of the census were used to examine a set of potentially significant parameters and to understand their role in defining the landslide dam hazard. The study area is considered representative of the modalities within which the phenomenon occurs.

## 2 Study area

**Figure 1:**
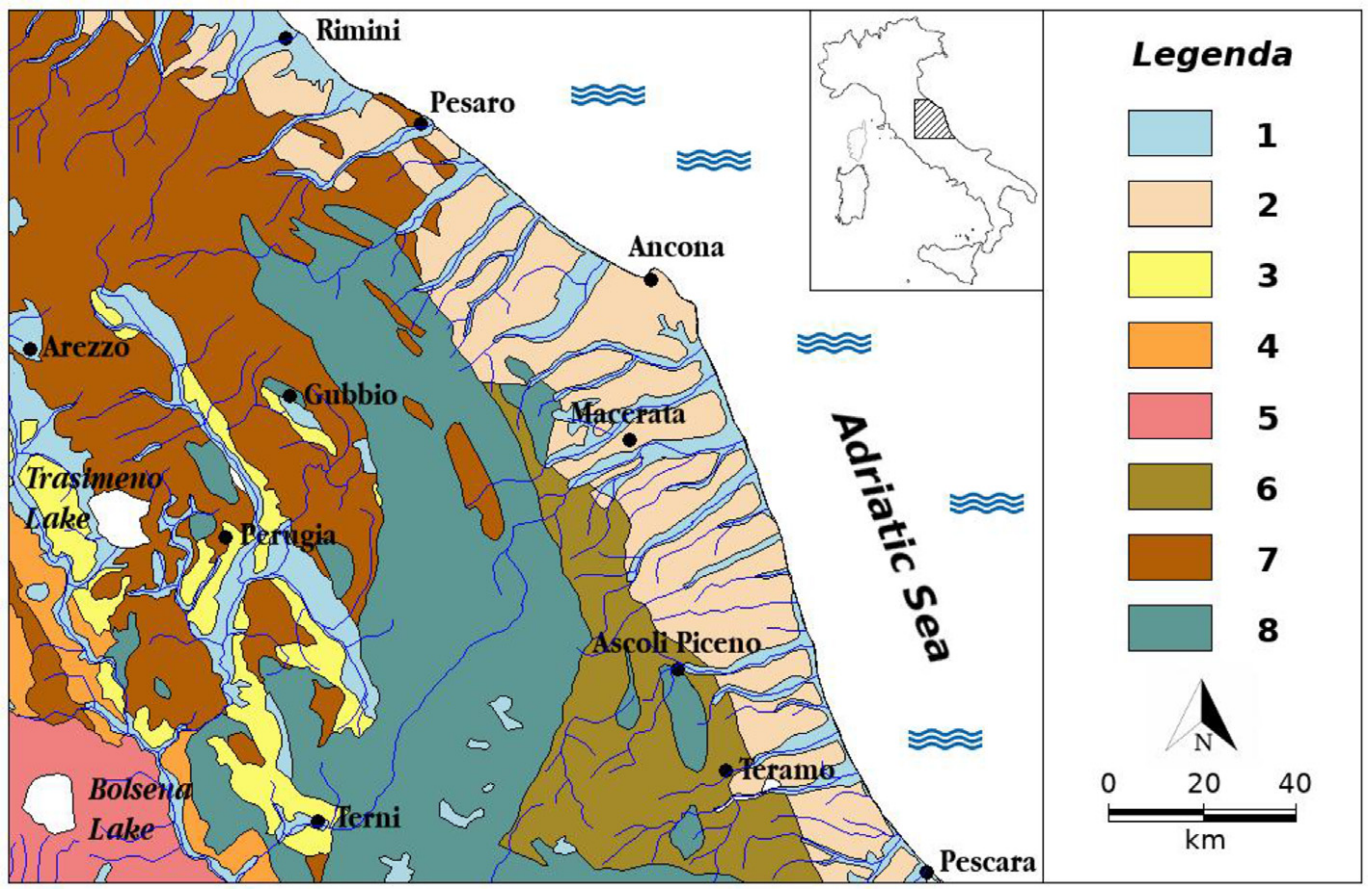
Geological sketch of study area. Legenda: 1. Present and recent alluvial and coastal sediments, also terraced (Olocene - Upper Pleistocene); 2. Foredeep clastic sediments, predominantly conglomerates, sands and clays (Pleistocene - Pliocene); 3. Clays, sands and conglomerates in continental facies, lacustrine and fluvial-lacustrine (Pleistocene - Pliocene); 4. marine sediments, predominantly clays, sands and conglomerates (Pleistocene - Pliocene); 5. Volcanites of Vulsini volcanic complex (Pleistocene); 6. Arenaceous and marly-arenaceous turbidites (Laga Flysch) (Upper Miocene); 7. Arenaceous and marly-arenaceous turbidites of Tuscan Nappe and Umbrian domain (Middle Miocene - Oligocene); 8. Limestones and marly limestones of Umbrian-Marchean Series (Oligocene-Trias).

Figure 1 shows the area under investigation, within a geological outline of the Italian peninsula. The area includes the whole of Umbrian-Marche Apennines and its boundaries: the tectonic contact area with the flyschoid units of the Marchean Foredeep (to the east); the pre-Apennine area (to the west) where continental and marine post-orogenic Plio-Pleistocene sediments outcrop and where the Tuscan Nappe currently contacts tectonically with the Umbrian domain.

The area under investigation thus partially includes the regions of Emilia-Romagna and Tuscany (in the north) and Latium and Abruzzo (in the south). These have close analogies, from the lithological-formational, geological-structural and geomorphological point of view, with the Umbria-Marche territory. Essentially, the area of investigation allows for the widest possible set of landslide dam case studies within an area representative of the greater central-northern Apennines. Examination of the census cases, the past examples of interference by slope processes on the dynamics of riverbeds, was expected to permit some conclusions about the predictability of this phenomenon.

## 3 Method: census of landslides interfering with fluvial dynamics in Apenninic Umbrian-Marchean and neighbouring areas

Landslide dam databases have been produced in Italy previously: Pirocchi (1991) for the Alpine area; Canuti et al. (1998) for the Northern Apennine; and Tacconi Stefanelli et al. (2015) for the whole national territory.

**Figure 2:**
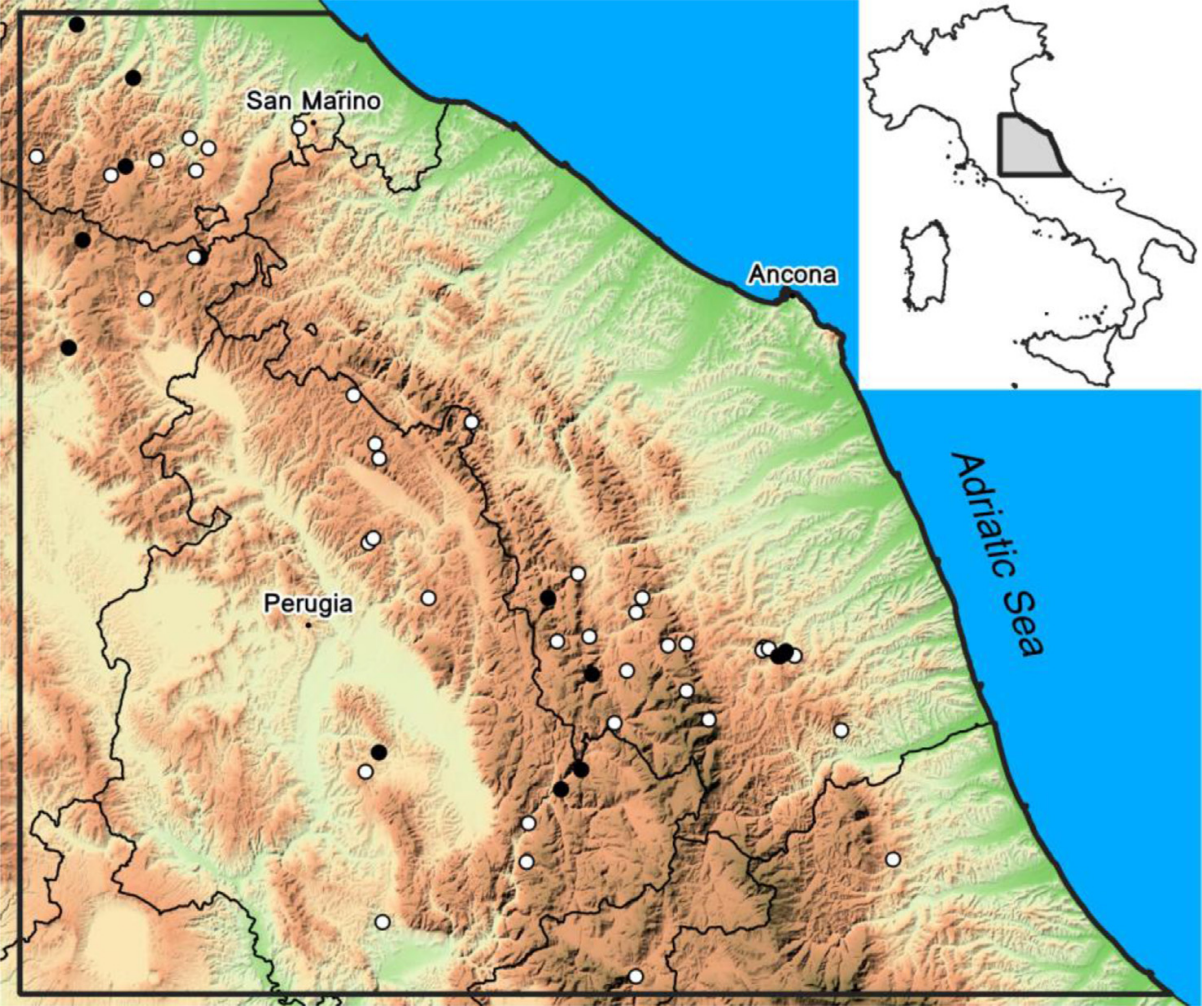
Localization of the 53 cases surveyed in the investigated area. White circles indicate landslide dams with formation of a dam lake; black circles indicate landslides which produced a partial occlusion and interfered with fluvial dynamics.

Starting from the known data, a relational database was developed; this was then extended, to include all the landslides, in the study area, that have interfered with riverbed dynamics, causing partial or total occlusion, in protohistoric, historical and recent times. The main sources used were the IFFI catalogue (Inventario dei Fenomeni Franosi in Italia, i.e. Landslides Inventory in Italy, http://www.isprambiente.gov.it/it/progetti/suolo-e-territorio-1/iffi-inventario-dei-fenomeni-franosi-in-italia) and the maps of the PAI (Piani di Assetto Idrogeologico, i.e. Hydrogeological Arrangement Plans, produced by Italian Regional Administrations). Data from the direct knowledge of the authors, bibliographic sources and specific geomorphological surveys, both on the ground and through the photo interpretation of the study area, were added. The number of landslides so identified (Figure 2) is significantly higher than in other known inventories, reaching 53 cases in total within the investigated area.

The landslides recorded include both cases in which the landslide did not completely occlude the riverbed, but did significantly interfere with the dynamics of the same (14 cases), and cases of total occlusion of the riverbed–alluvial plain system (39 cases).

The landslides have been codified by completing a "census form", with the aim of compiling in a single document the main factors influencing the phenomenon of the damming of a riverbed by landslide. The collected data were then used to create a database, a useful reference in the processing of statistical data. The census form had eleven sections, each divided into subsections, in order to provide a comprehensive framework of each event. The items are variously *free text*, *numeric*, *multiple choice* (where you can select multiple entries from a list of options) or *single choice* (where a single option is chosen).

The sections are:

• General cognitive data

This section includes the information that locates the landslide in space and time. The spatial data are numeric and free text and detail the location, relative to the WGS84 datum, using IGMI (Italian Military Geographical Institute) cartographic references. The temporal collocation of the event is also recorded.

• Morphological data

The morphological data indicate the main morphometric parameters that characterize the affected area. Generalised data are recorded initially (characteristics of the valley and the slope affected by landslide, area of the river basin subtended by the damming section), followed by a more detailed description of the riverbed affected by the occlusion and the landslide area. The field is single choice for the characteristics of the valley, numeric and free text for the other subsections.

• Geological data

This section contains the data on lithological-formational character and on the geotechnical properties of the rocks affected by landslide. The free text fields describe the geological formations affected by the landslide. The items “formation 1” and “formation 2” refer to cases where more than one geological formation is involved. The disposition of the rock masses and the land use on the slope on which the landslide was triggered are also recorded.

• Landslide classification

The classification by Cruden & Varnes (WP/WLI, 1993) is used here. The subsections describe the state, distribution, style of the activity and type of movement of each landslide, as well as the speed of the movement and its presumed duration (these values were assessed by the authors, and also used in the classification of landslides based on speed).

• Natural damming classification

The section describes the type of barrier, as taken from the classification of Costa and Schuster (1988), and its dimensions. Six types of accumulations are distinguished according to their geometric shape and the way they interfere with the riverbed. These are:

➢ ***Type I*** - The landslide body is small compared to the width of the riverbed–alluvial plain system. The channel blocking does not span the whole valley, allowing a diversion of the river course, which can quickly circumvent the barrier.
➢ ***Type II*** - In this case the barrier is large and occupies the entire valley floor. In some situations, the material that reaches the far side of the valley still has sufficient energy to a short distance up the opposite slope.
➢ ***Type III*** - The landslide causes complete closure of the valley floor and, in addition, the material spreads both upstream and downstream, potentially damming tributaries. This type of barrier typically involves large volumes of material.
➢ ***Type IV*** - The barrier is formed by the simultaneous collapse of material from both sides of the valley. Occlusion can be produced either by mid-valley fusion of the two landslides or by side by side juxtaposition of the landslide materials.
➢ ***Type V*** - The form of the barrier is characterized by a number of lobes that extend into the valley floor. The riverbed can then be blocked at several points and a series of small landslide dam lakes can be formed. These landslides are generally of the flow type.
➢ ***Type VI*** - The sliding surface of the landslide extends under the riverbed and re-emerges on the opposite side of the valley. The consequent uplift of the valley floor stops flow in the river channel and causes water to build up.

• Predisposing factors and triggering causes

The section includes information on predisposing factors and landslide triggers. Each subsection identifies a particular group of factors and causes (e.g., natural or anthropic, fixed or variable, etc.) that have triggered or favoured the occurrence of the landslide.

• Description of the phenomenon

This section describes the current conditions of the natural dam and the lake.

• The produced damages

Two columns of multiple choice symbols indicate (a) the damage caused directly by the landslide, from the moment it starts until it stops, and (b) the damage caused by the lake, because of upstream flooding or as a result of release of water in the case of sudden dam collapse.

• Implemented interventions

“Implemented interventions” includes all those works that have been carried out after the event to mitigate the damages, and reduce immediate risks, caused by the landslide and the occlusion.

• Cost of the interventions

This section records the expected or actual expenses (if known), incurred in implementing the aforementioned mitigation measures.

• Sources of knowledge - References

Sources of knowledge include the papers or reports, dealing with the case in question, used during the research phase.

In order to archive the data collected during the census, a relational database was created, using the “Base” tool of the open-source software “OpenOffice” (http://www.openoffice.org/).

The collected data were distributed in nine tables:

- General data
- Morphological data_Landslide Area
- Morphologica data_Valley Characteristics
- Morphological data_Stream
- Geological Data
- Landslide Classification
- Description of phenomenon
- Causes
- Damages_Interventions

**Figure 3:**
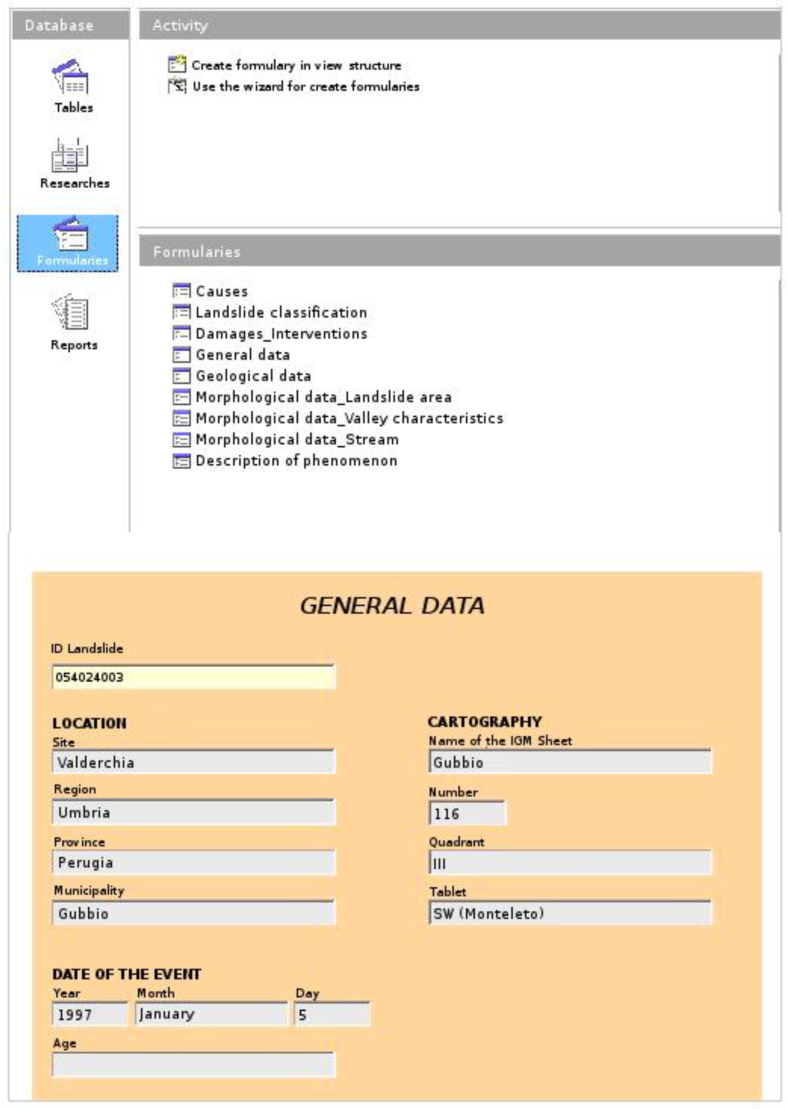
The database input forms produced using OpenOffice, with detail of the “General Data” section.

To facilitate data entry, “data entry forms” were created for each table. These are called *forms* in OpenOffice and the *form* for General Data is shown in Figure 3.

The numeric *ID Landslide* field identifies the *Primary Key*. For example:

ID Landslide: 54024003

• *54*: identifies the province in which the phenomenon occurred;
• *024*: identifies the municipality in which the phenomenon occurred;
• *003:* identifies that this is the third instance recorded within this municipality.

The first two entries (province and municipality) use the administrative codes attributed by ISTAT (Italian National Institute of Statistics - https://www.istat.it/it/archivio/6789).

**Figure 4:**
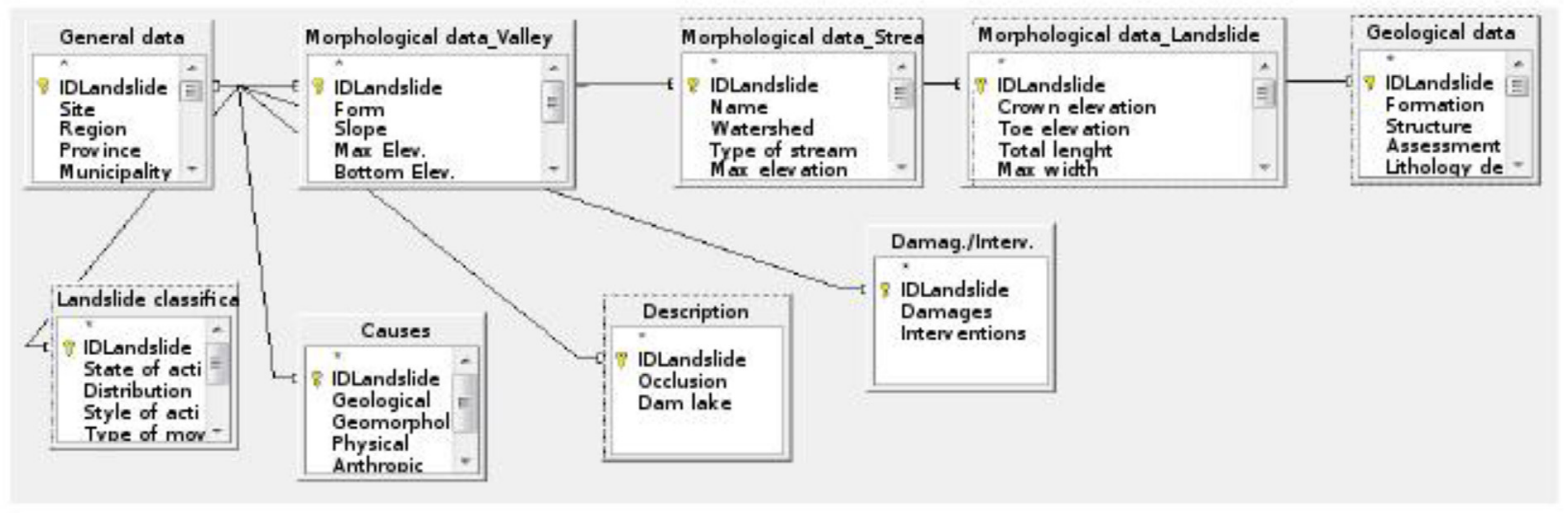
Relations between the different tables of the database.

In order to facilitate data entry, several summary tables, related by their ID were created. The type of relationship used was “one to one” (Figure 4). Data output used the OpenOffice report creation facility.

## 4 Data

### 4.1 The cases examined

In Umbria, which is where the census began, 13 events, with recent historical information and with clear morphological and hydrological evidence in the streams, were examined. Twelve of these are located in the province of Perugia: two in the River Puglia basin, i.e. Barattano (Cencetti and Conversini, 2003) and Fontivecchie; two in the River Ventia basin, i.e. C.se Sterpaiolo and Carpiano (Cencetti et al., 2015b); two in the River Burano basin, i.e. C. Buttafuoco and Pian Martino; one in the River Assino basin, i.e. Valderchia (Cencetti et al., 1998); one in the River Chiascio basin, i.e. Porziano; and four in the River Nera basin, i.e. San Lazzaro, Triponzo, Pian delle Melette (Barchi et al., 1993) and Piedipaterno (Conversini et al., 2005). Nearly all these rivers are in the basin of the Tiber River, flowing towards the Tyrrhenian Sea. The exception is the Burano River which is a tributary of the Metauro River, flowing towards the Adriatic Sea.

One event (Colle Pizzuto, in the Cerreta Creek basin, a tributary of the Nera River - Cencetti et al., 1996) was located in the province of Terni, also within the Tiber River basin.

By enlarging the survey area to the neighbouring provinces (Arezzo in Tuscany, Rieti in Latium, Ancona, Ascoli Piceno, Macerata, Fermo, Pesaro-Urbino in the Marche, Forlì-Cesena, Rimini and the Republic of San Marino in Emilia-Romagna; Teramo in Abruzzo) the recorded cases rose to 53. From a geological-structural point of view, the expansion brought into consideration territories bounded by the Val Marecchia to the north, the Laga Basin to the south, the Chiana Valley - Paglia Valley to the west and the Adriatic Sea to the east.

Table 1 shows the salient data relating to each of the landslides surveyed. The specified geographical coordinates (WGS84) refer to the point where the riverbed occlusion (partial or total) occurred.

**Table 1:** Main characteristics of the landslides interfering with the fluvial dynamics and fluvial systems (riverbeds–alluvial plains) in the study area.

ID Landslide	Lat. (°)	Long. (°)	Region	Sheet (IGM)	Locality (Municipality, Province)	River	Main Basin	Width riverbed – alluvial plain system (m)	Landslide type	Volume (10^3^ m^3^)	Geologic Formation	Presence of dam lake	Persistence of dam lake	Age (year or period)	Area of the basin subtended by dam (km^2^)	Landslide dam classification (Costa & Schuster)
99025001	43.9341	12,4135	Emilia Romagna	108 I NE	Caitasso (presso Acquaviva, RSM)	San Marino	Marecchia	250	flow	1200	“Argille di Casa I Gessi”	Yes	filled	1854	40.00	II
40044001	43.9247	12.1632	Emilia Romagna	108 IV NE	Sorbano (Sarsina, FC)	Savio	Savio	225	slide	2500	“Marnoso-Arenacea Romagnola”	Yes	filled	161	309.00	II
40009001	44.0277	12.0393	Emilia Romagna	100 III SW	Voltre (Civitella di Romagna, FC)	Voltre	Bidente/Ronco	250	slump/flow	100	“Marnoso-Arenacea Romagnola”	No	not formed	2013	25.00	
40011001	44.1191	11.9147	Emilia Romagna	99 II NE	Pezzolo di Sotto - Canova Domigiolo (Dovadola, FC)	Rio Pezzolo	Montone	65	slide	1720	“Marnoso-Arenacea Romagnola”	No	not formed	2005	3.00	
40001001	43.8683	11.9804	Emilia Romagna	108 IV SW	San Piero in Bagno (Bagno di Romagna, FC)	Savio	Savio	175	slump/flow	4500	colluvium of “Marnoso-Arenacea Romagnola”	Yes	drained	1855	81.55	IV
40001002	43.8819	12.0144	Emilia Romagna	108 IV SW	Tozzi (Bagno di Romagna, FC)	Savio	Savio	175	slide	3000	“Marnoso-Arenacea Romagnola”	No	not formed	1903	110.00	
40044001	43.8902	12.0858	Emilia Romagna	108 IV SE	Quarto (Sarsina, FC)	Savio	Savio	110	slump	16000	“Marnoso-Arenacea Romagnola”	Yes	existing (artificially maintained - reservoir)	1812	214.80	III
99026001	43.8707	12.1746	Emilia Romagna	108 I SW IV SE	Monte Ercole (Sant’Agata Feltria, RN)	Marecchiola	Fanante/Savio	50	flow	8000	“Ghioli di letto/Marnoso-Arenacea Romagnola”	Yes	drained	1561	15.10	III
40043001	43.9032	11.8118	Emilia Romagna	107 I SW	Corniolo (Santa Sofia, FC)	Bidente di Corniolo	Bidente/Ronco	80	slide	1000	“Marnoso-Arenacea Romagnola”	Yes	existing	1992	50.00	II
43007001	43.1324	13.1434	Marche	124 III NE	Campolarzo (Camerino, MC)	Chienti	Chienti	300	flow	8000	colluvium	Yes	drained	Olocene/Upper Pleistocene	268.30	I
43007002	43.1091	13.1282	Marche	124 III NE	Valdiea (Camerino, MC)	Chienti	Chienti	150	slide	500	“Scaglia bianca”	Yes	drained	Upper Pleistocene	224.00	I
43017001	43.0517	13.1962	Marche	124 III SE	Podalla (Fiastra, MC)	Rio Bagno	Fiastrone/Chienti	<10	slide	6000	“Maiolica”/“Marne a Fucoidi”	Yes	drained	Olocene/Upper Pleistocene	2.50	II
43018001	43.0135	13.1008	Marche	124 III SW	Nemi (Fiordimonte, MC)	Macereto	Chienti	110	flow	170	colluvium	Yes	drained	Olocene	20.75	II
43005001	42.9754	13.2325	Marche	132 I NW	Bolognola (Bolognola, MC)	Fiastrone	Fiastrone/Chienti	20	slide	3700	“Calcari diasprini”	Yes	drained	1930	4.95	VI
109015001	42.9260	13.2789	Marche	132 I NW	Gole dell’Infernaccio (Montefortino, FM)	Tenna	Tenna	28	fall/debris flow	80	“Scaglia rossa”	Yes	existing	2016	28.30	II
43027001	43.0101	13.0218	Marche	124 III SW	M. Uschio (Monte Cavallo, MC)	Vallicello	Chienti	30	slump/flow	1000	colluvium	No	not formed	Olocene	2.80	
43032001	43.0407	13.4118	Marche	124 II SE	Casa Carassa (Monte San Martino, MC)	Tennacola	Tenna	110	slump/flow	60	“Laga Flysch”	Yes	drained	1970/1980	95.00	III
43035001	43.0360	13.4075	Marche	124 II SE	Casa Carassa 2 (Penna San Giovanni, MC)	Fosso del Lago	Tennacola/Tenna	10–50	slump/flow	40	“Laga Flysch”	Yes	drained	1895	16.50	I
43035002	43.0383	13.4219	Marche	124 II SE	C. Guglielmi (Penna San Giovanni, MC)	Tennacola	Tenna	75	slump/flow	100	“Laga Flysch”	Yes	drained	1985	97.56	I
43032004	43.0248	13.4425	Marche	124 II SE	Molino I (Monte San Martino, MC)	Tenna	Tenna	50	slump/flow	70	“Laga Flysch”	Yes	drained	1994	150.12	I
43032005	43.0249	13.4467	Marche	124 II SE	Molino II (Monte San Martino, MC)	Tenna	Tenna	50	slide	585	“Laga Flysch”	No	not formed	1978	148.00	
43032006	43.0289	13.4572	Marche	125 III SW	C. Vita (Monte San Martino, MC)	Tenna	Tenna	65	slide	400	“Laga Flysch”	No	not formed	1980	156.00	
43032007	43.0322	13.4599	Marche	125 III SW	Railway Station of Santa Vittoria in Matenano (Monte San Martino, MC)	Tenna	Tenna	125	slump/flow	320	“Laga Flysch”	No	not formed	1975	163.00	
43052002	43.0728	13.0198	Marche	124 III SW	Sasso Piano (Serravalle del Chienti, MC)	Chienti di Gelagna	Chienti	125	slide	3000	“Scaglia bianca”/“Marne a Fucoidi”	Yes	drained	Olocene/Upper Pleistocene	91.00	II
43039001	43.1773	13.0016	Marche	123 I SE	Ormagnano (Pioraco, MC)	Potenza	Potenza	215	flow	3000	colluvium	Yes	drained	Olocene/Upper Pleistocene	171.50	IV
43050001	43.1402	12.9309	Marche	123 II NE	Monte Vermenone (Sefro, MC)	Scarzito	Potenza	45	flow	150	colluvium	No	not formed (prevented)	1997	4.77	
43052001	43.0675	12.9471	Marche	123 II SE	Castello di Serravalle (Serravalle del Chienti, MC)	Chienti di Gelagna	Chienti	130	slide/flow	1000	“Marne a Fucoidi”/“Scaglia rossa”	Yes	drained	1279	54.00	III
42044001	43.4362	12.7771	Marche	116 II NW	Montelago (Sassoferrato, AN)	Fosso del Lago	Esino	140	slide	6000	“Marne a Fucoidi”	Yes	filled	7000-5000 b.P.	2.00	VI
43011001	43.0528	13.2377	Marche	124 II SW	Campo Lupo (Cessapalombo, MC)	Rio del Monte	Fiastrone/Chienti	45	slump/flow	340	“Scaglia rossa”/“Scaglia cinerea”	Yes	existing	2006–2012	2.00	II
109036001	43.0241	13.4790	Marche	125 III SW	Santa Vittoria in Matenano (Santa Vittoria in Matenano, FM)	Lame	Tenna	65	slide/flow	10	“Laga Flysch”	Yes	drained	1985	32.00	II
44007001	42.8969	13.5746	Marche	133 IV SE	Porchiano (Ascoli Piceno, AP)	Chiaro Morto	Chiaro/Tronto	<10	slide	1700	“Laga Flysch”	Yes	existing	2014	3.51	II
43057001	42.9282	13.0672	Marche	132 IVI SE	Visso (Visso, MC)	Nera	Nera/Tevere	40	slide//flow	70	“Maiolica”	Yes	existing	2016	141.00	III
99026002	43.9069	12.2052	Marche	108 I SW IV SE	C. Frassino (Novafeltria, RN)	Fanante	Savio	75	slump	2500	“Gessoso-Solfifera”	Yes	filled	1855	13.17	III
51006001	43.5855	11.8702	Tuscany	114 I SE	Le Mottacce (Capolona, AR)	Arno	Arno	100	slump/flow	90	“Arenarie del Cervarola”	No	not formed	1987	739.00	
51003001	43.7266	12.1768	Tuscany	108 III SE	San Patrignano (Badia Tedalda, AR)	Marecchia	Marecchia	200	slide	100	“Monte Morello”	No	not formed (prevented)	1990	41.40	
51003002	43.7280	12.1637	Tuscany	108 III SE	Tramarecchia (Badia Tedalda, AR)	Marecchia	Marecchia	125	slide//flow	1500	“Monte Morello”	Yes	filled	1945	40.80	II
51015001	43.7626	11.9105	Tuscany	107 II NE	Frassineta (Chiusi della Verna, AR)	Fosso del Rimaggio	Corsalone/Arno	50	slump/flow	450	“Monte Morello”	No	not formed	1992	1.20	
51030001	43.6618	12.0494	Tuscany	108 III SW	Belmonte (Pieve Santo Stefano, AR)	Tevere	Tevere	400	slump/flow	4500	“Monte Morello”	Yes	artificially drained	1855	106.90	III
54001001	43.1485	12.6620	Umbria	123 III NE	Porziano (Assisi, PG)	Rio Grande	Chiascio/Tevere	10–50	slump/flow	60	“Marnoso-Arenacea”	Yes	filled	1963	3.24	II
54010001	42.8228	12.9400	Umbria	131 II NE	Triponzo (Cerreto di Spoleto, PG)	Corno	Nera/Tevere	75	flow	3	travertine	No	not formed	1986	510.00	
54022001	42.8663	12.5038	Umbria	131 IV SW	Barattano (Gualdo Cattaneo, PG)	Puglia di Barattano	Puglia/Tevere	<10	slump/flow	30	Sediments of “Bastardo Basin” (Villafranchiano Auct.)	Yes	filled	1996	5.65	I
54022002	42.8972	12.5357	Umbria	131 IV SW	Fontivecchie (Gualdo Cattaneo, PG)	Puglia	Puglia/Tevere	<10	slump	10	Sediments of “Bastardo Basin” (Villafranchiano Auct.)	No	not formed	2006	6.00	
54024001	43.2514	12.5434	Umbria	123 IV NW	C.se Sterpaiolo (Gubbio, PG)	Ventia	Ventia/Tevere	50	slide	190	“Marnoso-Arenacea”	Yes	existing	1963	5.08	II
54024002	43.2441	12.5319	Umbria	123 IV SW	Carpiano (Gubbio, PG)	Carpiano	Ventia/Tevere	<10	slump	420	“Marnoso-Arenacea”	Yes	filled	Olocene/Upper Pleistocene	2.17	IV
54024003	43.3830	12.5641	Umbria	116 III SW	Valderchia (Gubbio, PG)	San Donato	Assino/Tevere	30	slump/flow	1000	“Marnoso-Arenacea”/“Schlier”	Yes	artificially drained	1997	4.20	II
54024004	43.4072	12.5567	Umbria	116 III SW	Pian Martino (Gubbio, PG)	Tributary of Fosso dei Tre Ponti	Burano/Candigliano/Metauro	<10	slump/flow	628	“Arenarie e Marne di M. Vicino”	Yes	existing	1960	19.36	V
54024005	43.4894	12.5125	Umbria	116 IV NW	C. Buttafuoco (Gubbio, PG)	Fosso di Burano	Burano/Candigliano/Metauro	120	slump/flow	6000	“Marnoso-Arenacea”	Yes	drained	Olocene	19.00	II
54045001	42.7056	12.8557	Umbria	131 II SE	Pian delle Melette (Sant’Anatolia di Narco, PG)	Fosso di Gavelli	Nera/Tevere	180	fall/debris flow	4000	“Maiolica”	Yes	filled	Olocene/Upper Pleistocene	14.00	II
54058001	42.7694	12.8637	Umbria	131 II SE	Piedipaterno (Vallo di Nera, PG)	Nera	Nera/Tevere	50	flow	8	colluvium	Yes	drained	1945	1121.00	II
55029001	42.6166	12.5276	Umbria	138 IV NW	Colle Pizzuto (Sangemini, TR)	Cerreta	Caldaro/Nera/Tevere	30	flow	1000	“Santa Maria di Ciciliano” (Villafranchiano Auct.)	Yes	existing	1960	0.76	II
54043001	42.8536	12.9874	Umbria	132 IV SW	San Lazzaro (Preci, PG)	San Lazzaro	Nera/Tevere	60	fall	150	“Scaglia rossa”	No	not formed	1997	9.75	
57057001	42.5080	13.0876	Latium	139 IV SE	Villa Camponeschi (Posta, RI)	Velino	Velino/Nera/Tevere	75	slump	2000	“Marne a Cerrogna”	Yes	filled	Olocene/Upper Pleistocene	165.20	I
67041001	42.6792	13.6749	Abruzzo	133 III SE	Mass.a Scapriano (Teramo, TE)	Fosso Grande	Vezzola/Tordino	70	slump/flow	2000	“Laga Flysch”	Yes	drained	1895	11.00	III

### 4.2 Parameters examined

The most important parameters of each event, as recorded in the individual census cards, were statistically processed. These main characteristics, further discussed below, were:

- Space-time collocation;
- Type of landslide material;
- Landslide type;
- Landslide volume;
- Width of the affected riverbed–alluvial plain system;
- Area of the basin subtended by the section of riverbed affected by the landslide;
- Type of the natural dam, based on the classification of Costa and Schuster (1988);
- Presence, or not, of a dam lake and its evolution.

#### 4.2.1 Space-time collocation

Nearly all the recorded landslides occurred within the ridge of the Umbria-Marche-Romagna Apennines. Among the 53 phenomena recorded, 16 occurred to the west of the main divide of the Apennines, in the Tyrrhenian drainage basins (River Tiber and River Arno), while the remaining 37 occurred in the East, in basins that flow into the Adriatic Sea (from Romagna to the Marche, and to Abruzzo).

**Figure 5:**
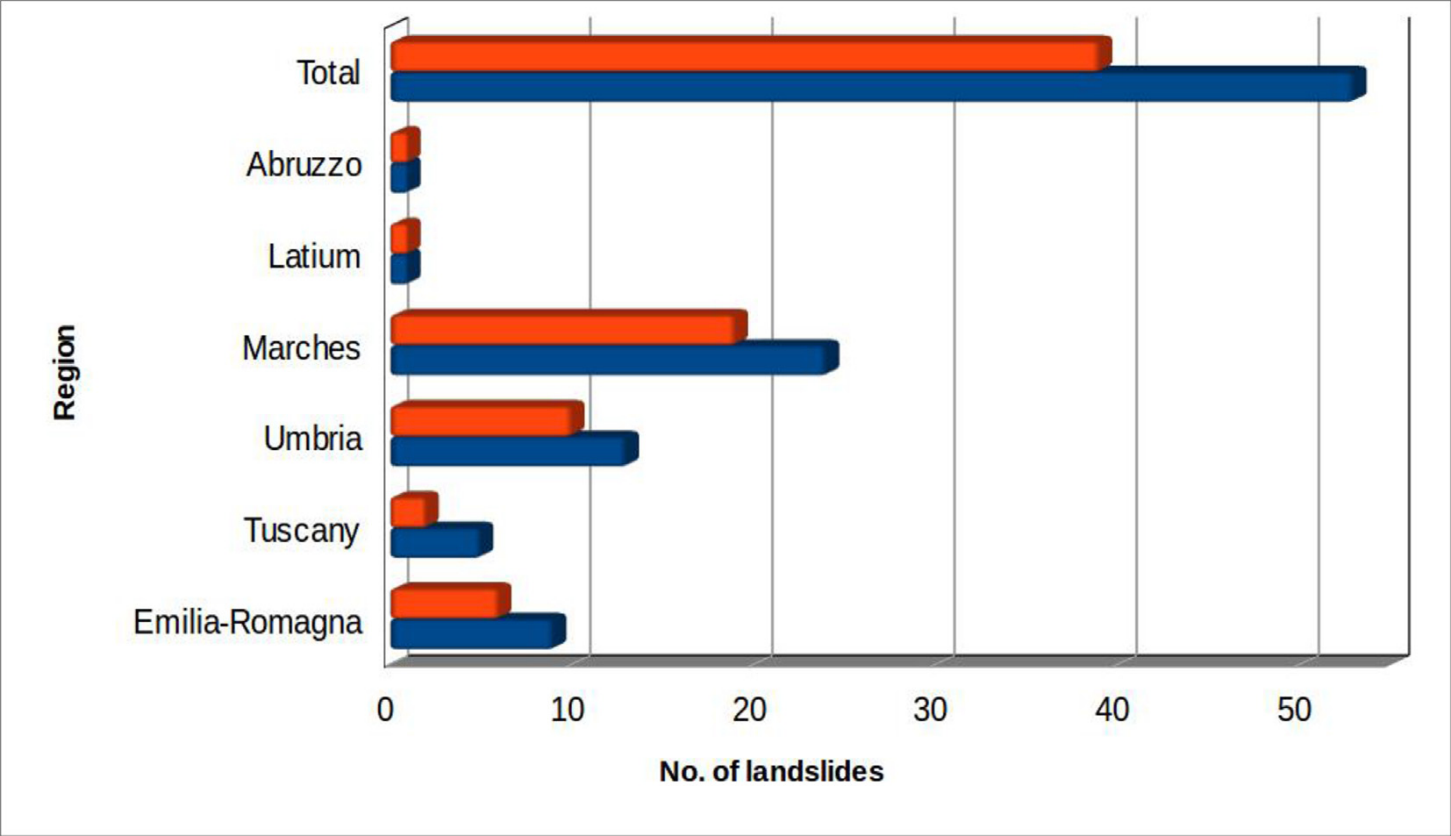
Administrative Italian Regions affected by landslide dams in the study area. The colour orange indicates landslides which produced dam lakes; all considered landslides are in blue.

**Figure 6:**
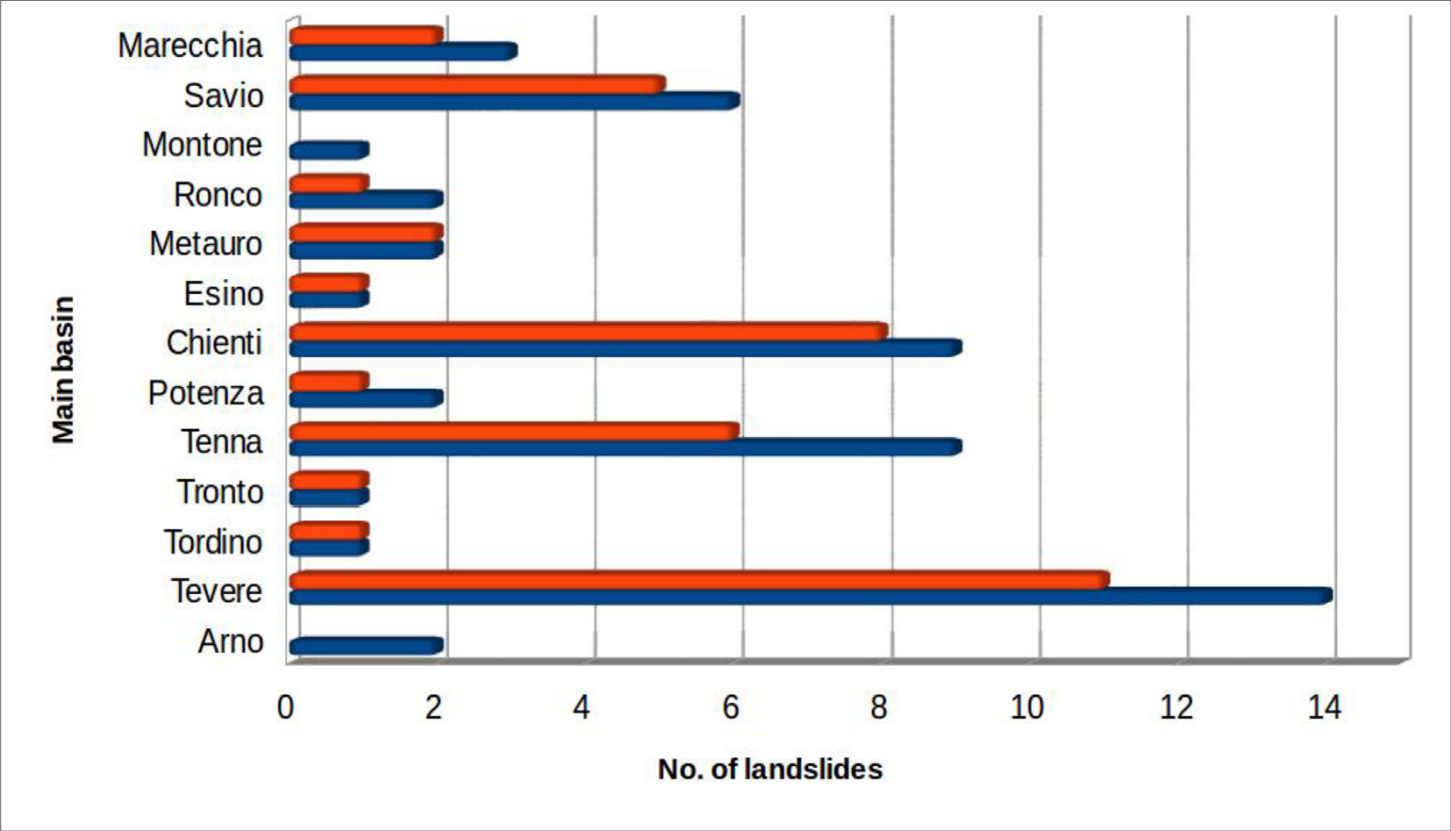
Main rivers basins affected by landslide dams in the study area. The colour orange indicates landslides which produced dam lakes; all considered landslides are in blue. All rivers are flowing towards Adriatic Sea, except the last ones (Tiber and Arno) that are flowing towards Tyrrhenian Sea.

Figure 5 shows the regional distribution of surveyed landslides. Figure 6 indicates the main rivers basins affected by the landslides.

This non-homogeneous distribution of landslides reflects the geological-structural situation that characterizes the studied area: on the Adriatic side of the Apennines, less stable pelitic rocks are very common. In addition, from a morphological-structural point of view, the Tyrrhenian hydrographic drainage network presents an angular pattern, while on the Adriatic side the rivers of the Marche show a general cataclinal pattern. In the latter, the thalweg has higher slopes, there are frequent and intense vertical erosion processes and high energy of relief. These factors produce, on the Adriatic side, a greater tendency to land sliding on the valley slopes and highly favourable conditions for landslide dam formation.

**Figure 7:**
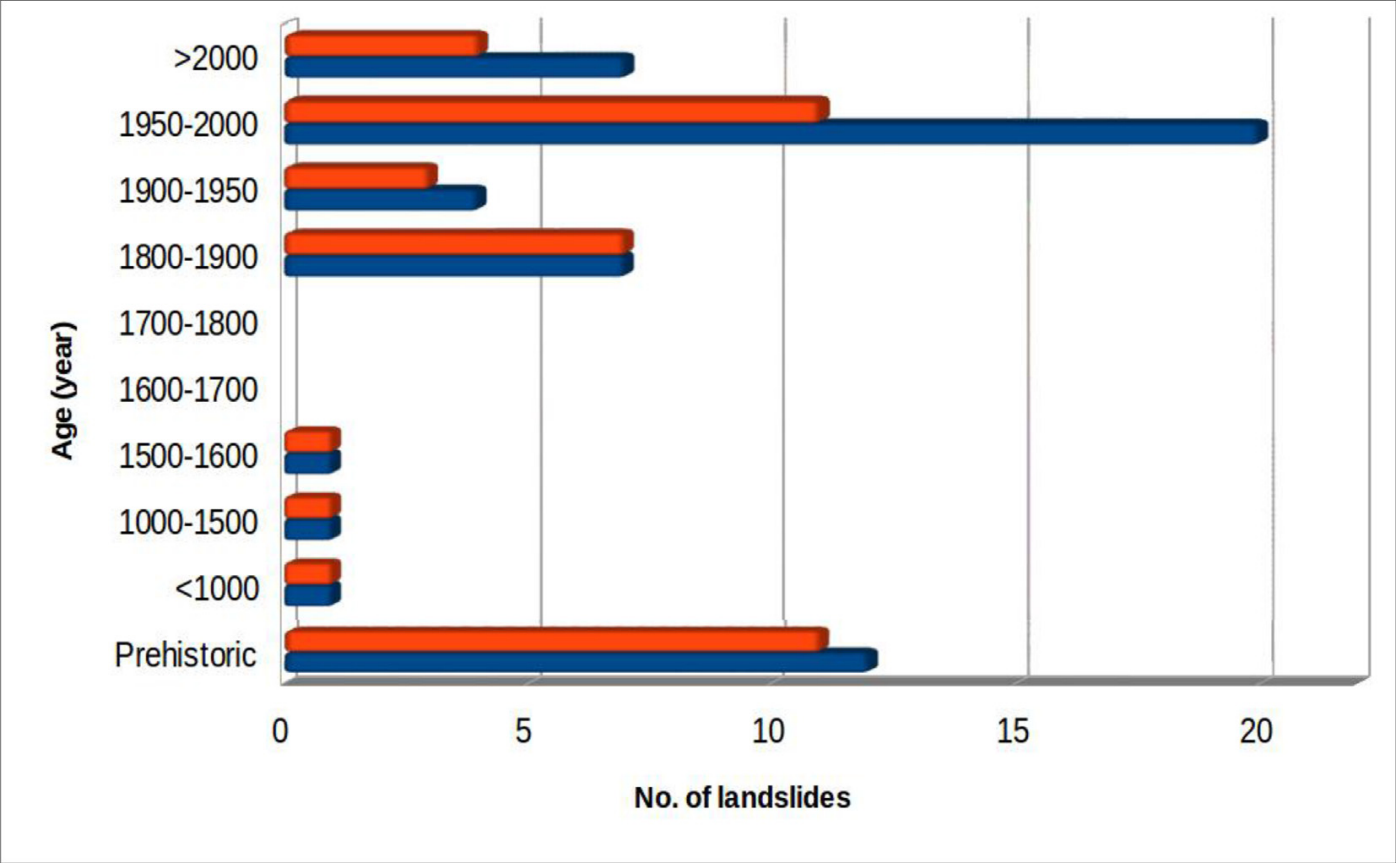
Temporal distribution of landslides interfering with riverbed dynamics in the study area. The colour orange indicates landslides which produced dam lakes; all considered landslides are in blue.

Figure 7 shows the temporal distribution of the recorded events.

The landslides referred to as “prehistoric” are derived from geomorphological features and the presence of lacustrine sediments, indicative of ancient dam lakes.

Precise historical references of some ancient landslides were found. Three of these affected the River Savio basin:

- the landslide at Sorbano, in Emilia Romagna, which in the 2nd century A.D. caused the formation of a lake on the Savio River, whose rapid filling inundated the Roman necropolis of Pian di Bezzo, in the province of Forlì-Cesena (Veggiani, 1954; Ortalli, 1997; Ortalli et al., 2008);
- the landslide at Monte Ercole, in the municipality of Sant'Agata Feltria (province of Rimini, in Emila-Romagna), which directly affected the town in 1561, causing much damage, including destruction of the palace of Duke Federico da Montefeltro and the Monastery of the Clarisse (Persi et al., 1993; Bisci et al., 1996);
- the landslide at Quarto (1812), the most impressive among those examined, involving approximately 16, 000, 000 m^3^ of material. The event was studied by Eng. Giovanni Bertoni who proposed a reconstruction of the events in 1843 (Bertoni, 1843). Eighteen people died as a result of the landslide that created a lake, still present, by damming of the Savio River. In the following century (1922), when the lake was nearly filled to capacity, the Hydroelectric Society of the Upper Savio (SIDAS) built, on the natural barrier, a dam and a hydroelectric plant. The current lake has an area of 85 ha and a capacity of about 9 million cubic meters.

Other historical landslides worthy of note include:

- the landslide that destroyed the Serravalle del Chienti Castle, in the province of Macerata, and occluded the Chienti River in 1279, as a result of a strong earthquake that affected the area of Camerino (Monachesi, 1987);
- the landslide that produced, in 1855, the total occlusion of the River Tiber. This Belmonte landslide, close to Pieve S. Stefano (province of Arezzo, Tuscany), caused the flooding of the Tuscan town and the death of 4 people (Mercanti, 1859; Cencetti and Viglione, 1995).

The majority of the other cases (about 58%) are concentrated in the period from 1900 to the present: this, however, does not imply a recent upsurge in occurrence, but simply that information about past events is very rare.

**Figure 8:**
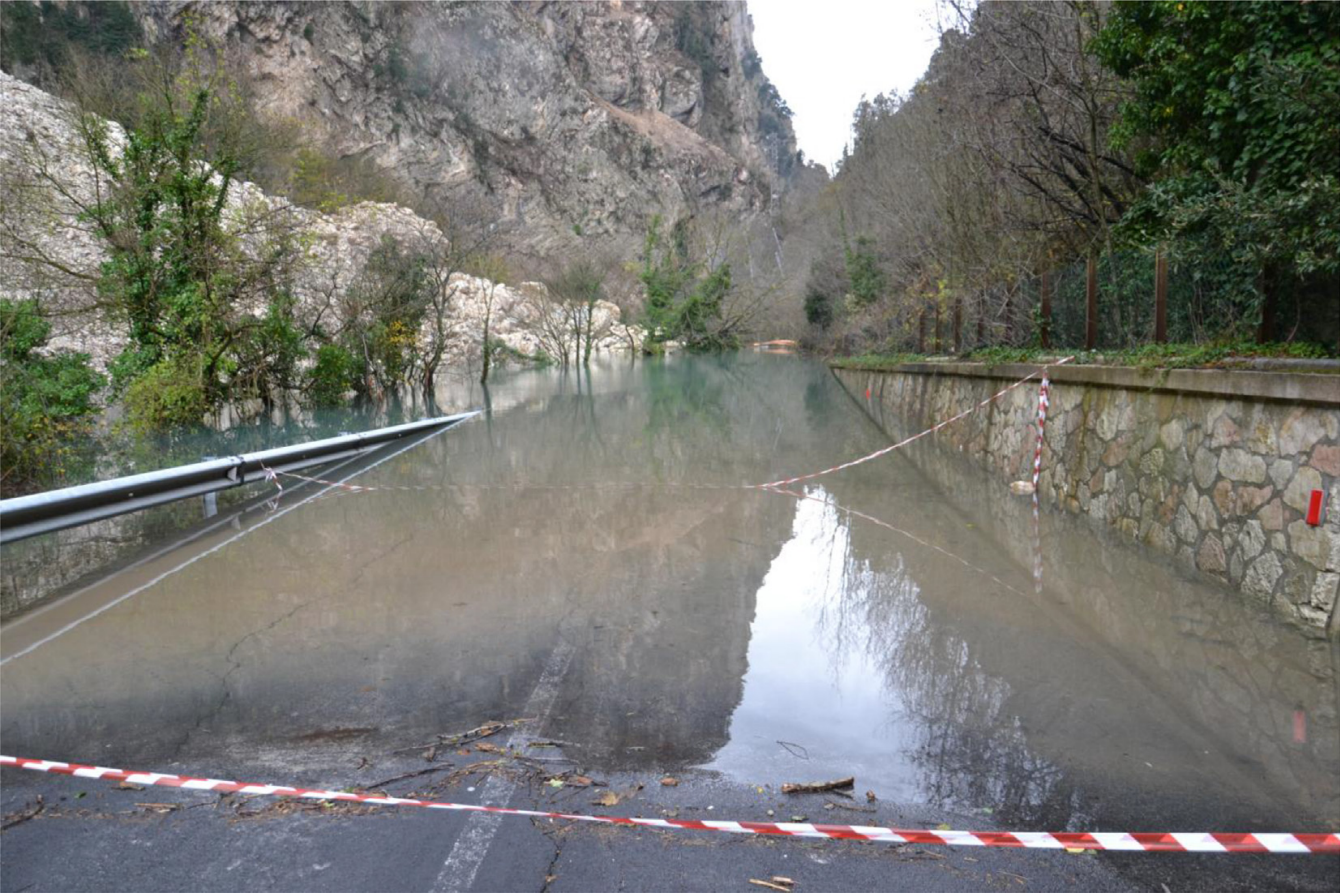
The seismic-induced landslide at Visso, which occluded the River Nera during the seismic event of October 30, 2016 (photo by Corrado Cencetti).

The two most recent landslide dams surveyed were seismic-induced in 2016: the landslide at Visso that obstructed the River Nera after a rock fall (Figure 8); and the landslide at the Infernaccio Gorges, on the Tenna River. Both followed the shock of October 30, 2016 (M_R_ 6.5) that affected the Marche region during the earthquake crisis of Umbria-Marche, responsible for the destruction of, among others, the communes of Accumoli and Amatrice (Romeo et al., 2017).

#### 4.2.2 Lithological characteristics of the slopes affected by landslides

Knowledge of the lithotype affected by the landslide is important for evaluation of the behaviour of the rocks when they interfere with the dynamics of riverbeds.

Evaluating parameters such as sediment grain-size and texture permits estimation, as a first approximation and in the absence of a specific characterization, of the values of some mechanical and geotechnical parameters of the landslide body constituting the barrier. These include permeability, the angle of internal friction, the angle of stability of the paraments of the natural dam and are important elements because they determine the stability and resistance of the dam itself.

**Figure 9:**
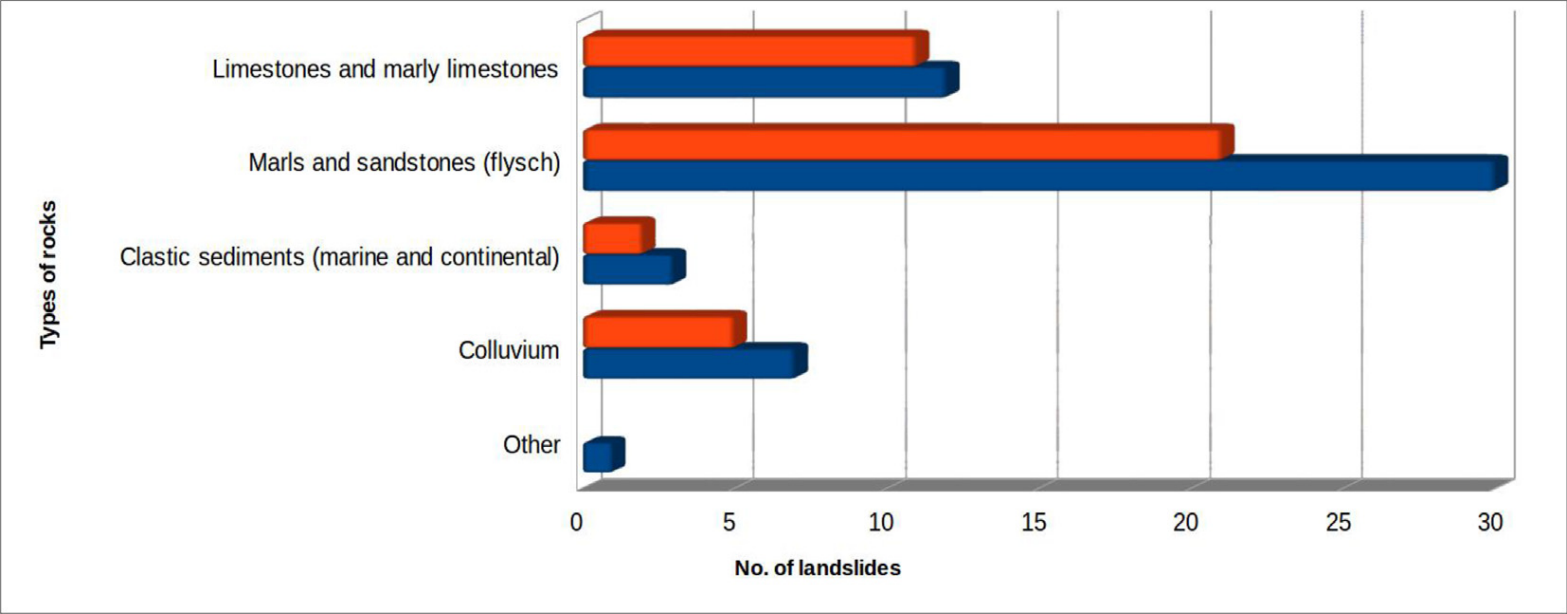
Distribution of landslides interfering with riverbed dynamics, based on the main types of rocks involved. The colour orange indicates landslides which produced dam lakes; all considered landslides are in blue.

In the study area, events that involved arenaceous-clayey-marly lithotypes in facies of flysch were clearly prevalent (56.6% of the total - Figure 9). Next most frequent were landslides on calcareous and calcareous-marly stratified rocks of the pelagic series (22.6%) and those on debris (13.2%). The post-orogenic sediments (Plio-Pleistocene), both in marine and continental facies (conglomerates, sands and clays), were involved in only 5.7% of cases. Evidently, the alternation of lithotypes with different geotechnical characteristics, sandstones and marls/clays in flysch facies, is a determining factor in the frequency of landslides.

#### 4.2.3 Landslide types

**Figure 10:**
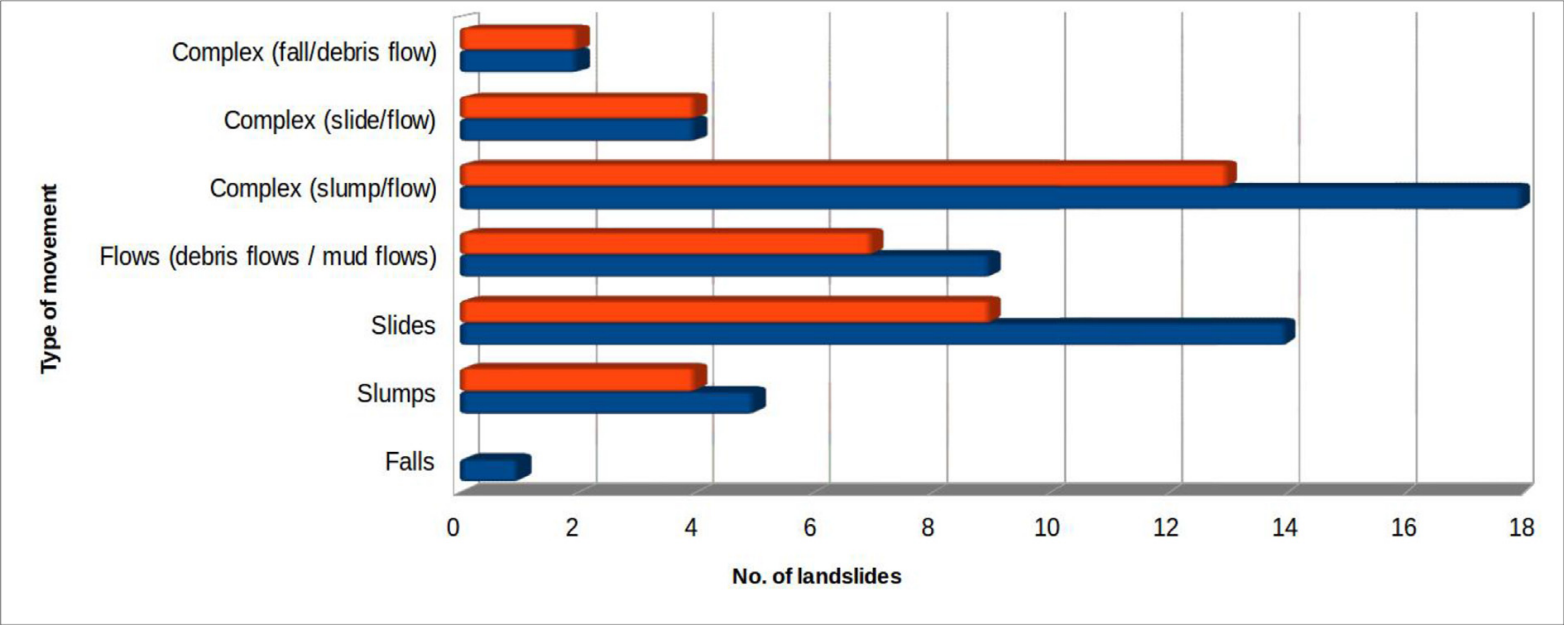
Type of landslides that have caused total occlusion of the riverbed. The colour orange indicates landslides which produced dam lakes; all considered landslides are in blue.

The type of landslide movement was based on UNESCO Working Party for World Landslide Inventory (WP/WLI) classifications (WP-WLI, 1993 - Figure 10). As most of the events occurred on marly-arenaceous flysch, it is logical to expect that the most frequent movements, that have produced the total occlusion of the riverbed-floodplain system, have been slides and slumps. These together represent 35.8% of the surveyed cases, and together with flows (17%) are often combined in complex landslides (41.5%). Therefore, these types of movement represent in total about 94% of the surveyed cases. A single case of fall occurred on the marly limestone of the "Scaglia Rossa" formation (landslide of San Lazzaro, near Preci, in Valnerina) which, however, did not result in total occlusion of the riverbed. Two other cases were the result of falls in which the landslide material, strongly fractured, was subsequently mobilized as debris flows, producing the riverbed occlusion: Pian delle Melette, near Sant'Anatolia di Narco, in Valcasana (rock avalanche - Barchi et al., 1993); and the landslide in the Infernaccio Gorges, near Montefortino, already mentioned, seismically induced by the earthquake of 30 October 2016 in the Umbria-Marche area.

#### 4.2.4 Landslides volumes

Calculation of the volume of surveyed landslides has proved useful in defining a classification, based on the order of magnitude of accumulations, to be used in statistical processing.

**Figure 11:**
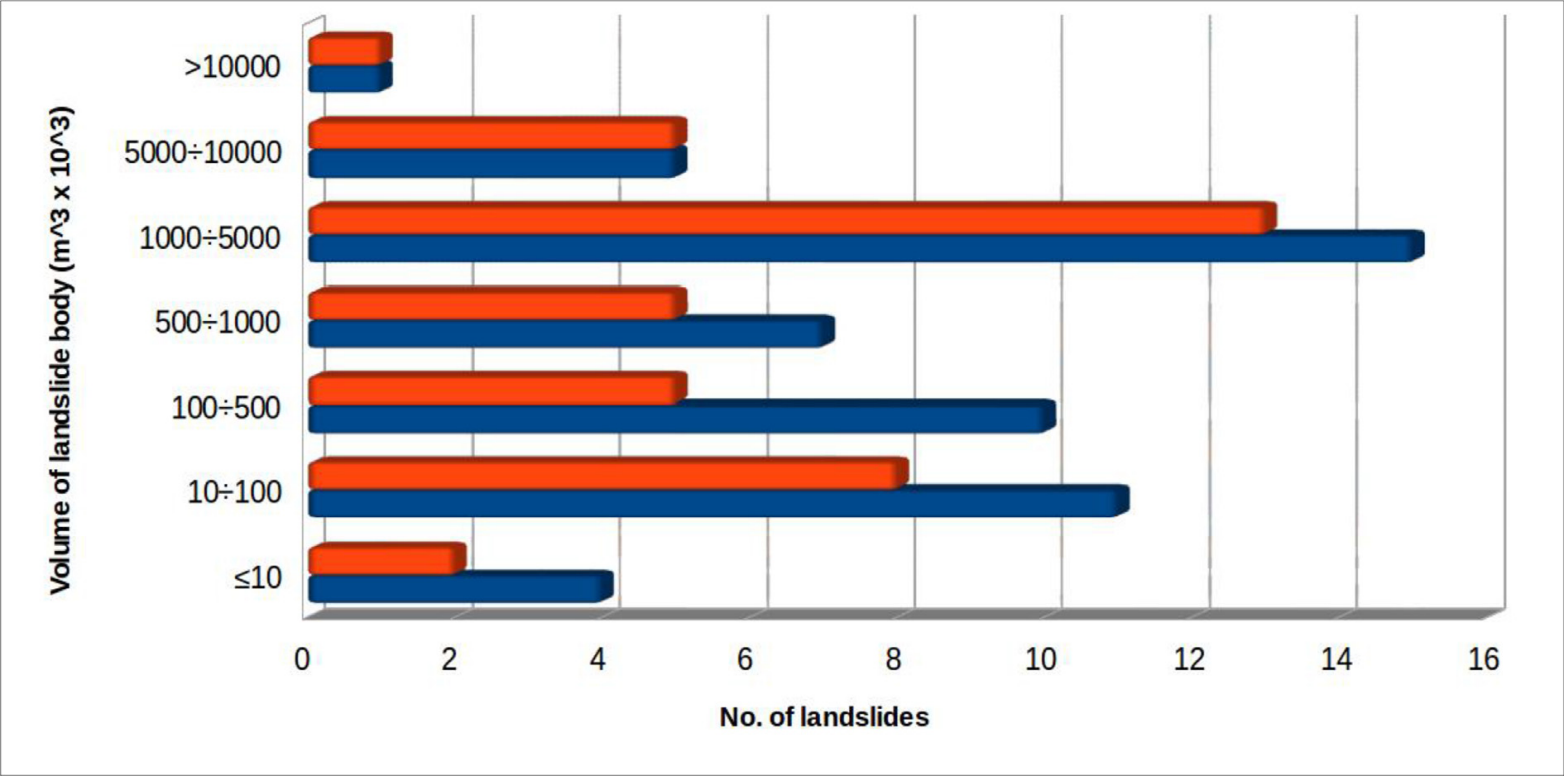
Volumes of landslides involving riverbed–alluvial plain systems. The colour orange indicates landslides which produced dam lakes; all considered landslides are in blue.

Scarcity of information, especially on the average depth of the sliding surface, creates uncertainty. In the absence of specific investigations, depth has often been very roughly approximated, using the available data. The volumes of surveyed landslides is however sufficiently reliable to define volume classes (Figure 11) covering landslide bodies whose volumes range from thousands of cubic meters to tens of millions of cubic meters.

The histogram shows that most cases of total river occlusion, with formation of a dam lake, involve a landslide volume of less than 5 million cubic meters (84.6%), while a third of the total occlusions (33.3%) involve volumes between 1 and 5 million cubic meters.

Among all the surveyed landslides, as could be expected, the percentage of those that produced riverbed total occlusion with the formation of a lake increases with increasing volume. The most impressive case in the examined area (the only one that exceeds 10 million m^3^) is that of the Quarto landslide, on the River Savio, whose estimated volume is about 16 million cubic meters.

#### 4.2.5 Width of the affected riverbed–alluvial plain system

**Figure 12:**
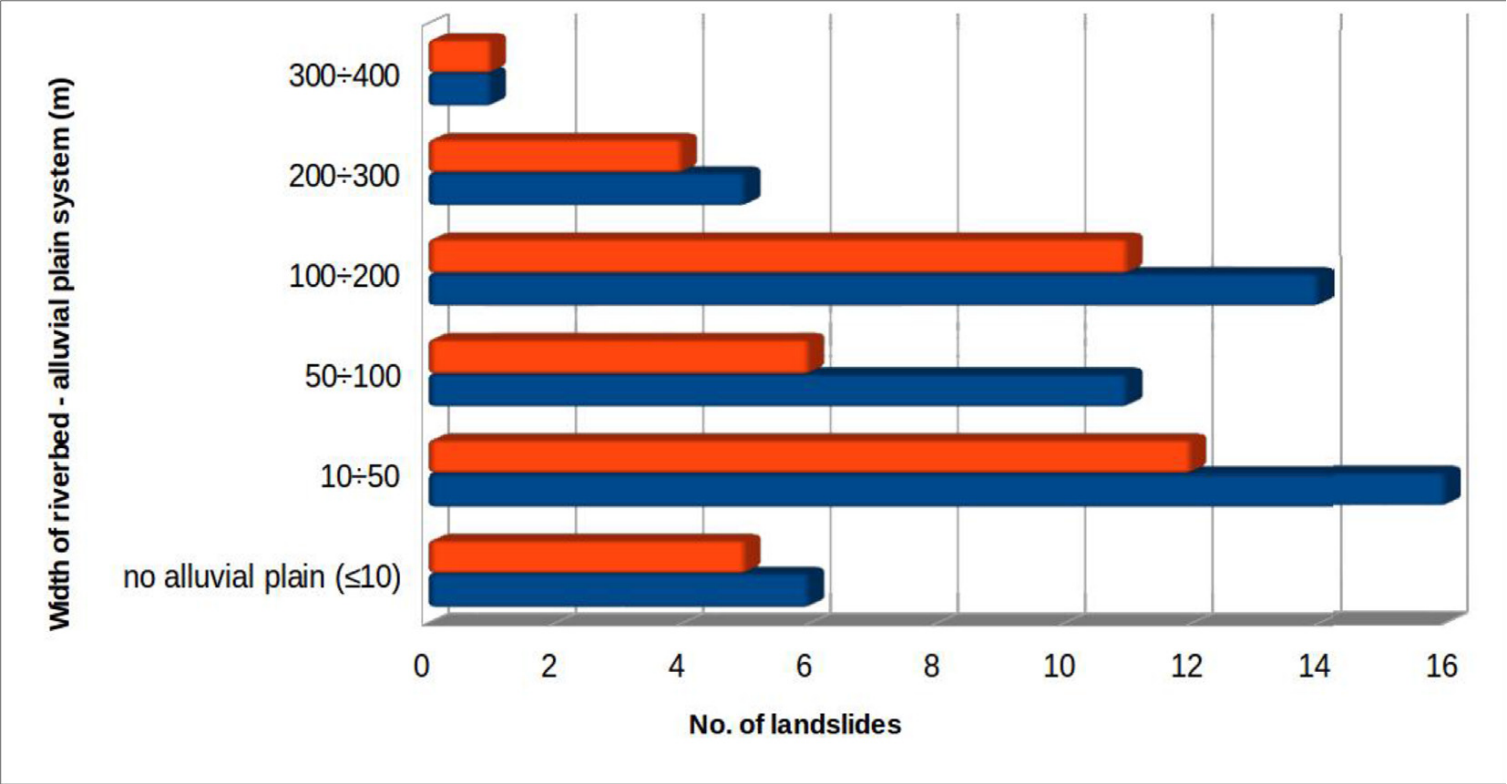
Width of the affected riverbed-alluvial plain systems. The colour orange indicates landslides which produced dam lakes; all considered landslides are in blue.

As for the previous parameter, width classes have been defined to aid analysis (Figure 12). The classes range from the situation of a fixed riverbed, in which the alluvial plain is absent or very little developed (<10 m), up to a width of 400 m.

In the study area, 41.5% of the riverbed obstructions, generally considered, occurred in situations where the width of the alluvial plain was less than 50 m, while 88.7% occurred where the width was under 200 m. The maximum width (400 m) was found for the landslide at Pieve S. Stefano, which completely occluded the Tiber Valley in 1855.

#### 4.2.6 Area of the river catchment upstream of the occlusion

**Figure 13:**
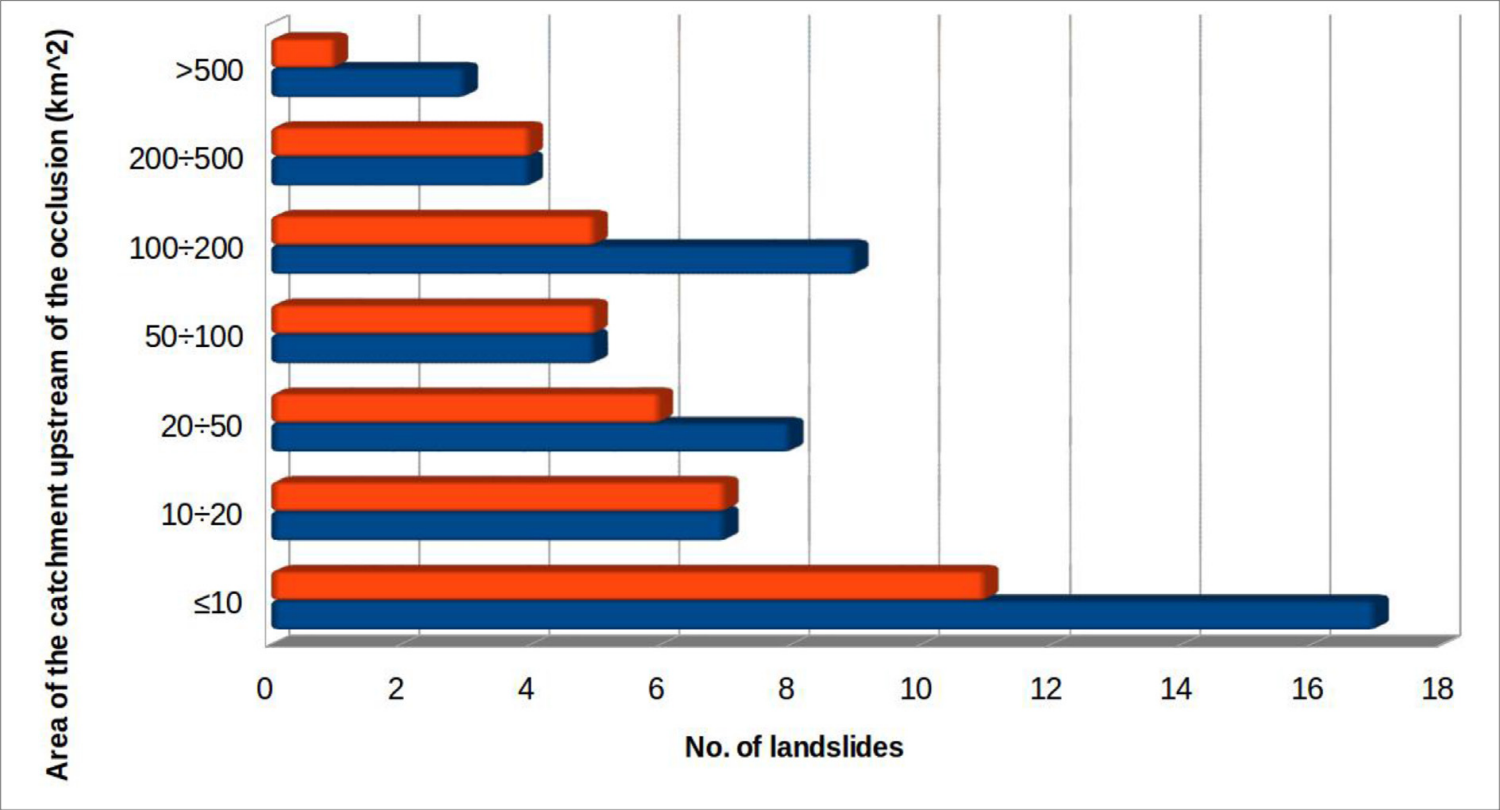
Distribution of landslides according to the area of the basin subtended by the section of the riverbed-alluvial plain systems involved. In blue all the considered landslide; in orange the landslide dams s.s. which produced the formation of a dam lake.

Another parameter considered, for all cases, was the area of the catchment upstream of the riverbed section affected by landslide (Figure 13).

A landslide that interferes with the fluvial system can occur in any section of riverbed along the stream. This gives the parameter a wide range of possible values which can arise from diverse geometries. However, the reaches of mountain valleys normally having small basins and minimal alluvial plains, are more vulnerable to being totally dammed. On the other hand, lower valley reaches, having a bigger basin and a more developed alluvial plain, are more difficult to dam.

This parameter has been included in the analysis because, in general, the larger the area of the river catchment upstream of the occlusion, the greater the discharge flow of the stream, which can affect:

- the possibility that the obstruction will generate a dam lake. In fact, high flow means high stream power (De Rosa et al., 2019), such that the material collapsed in the riverbed can be eroded more easily, preventing the formation of a dam. All this, of course, is a function of the magnitude of the landslide: if the landslide is large, or very fast, it will be able to block the stream, despite to the flow of the riverbed involved;
- the filling time of the dam lake, in the case of obstruction. The time to overflow of the landslide body depends on both the capacity of the formed lake and the incoming flow.

The analysis of the histograms shows, in fact, that more than half of the total surveyed occlusions (61.5%) occurred in situations in which the upstream river catchment had an area of less than 50 km^2^. Almost half of these (11 out of 24, or 45.8%) occurred in conditions in which the dam basin was less than 10^2^.

#### 4.2.7 Type of natural dam

**Figure 14:**
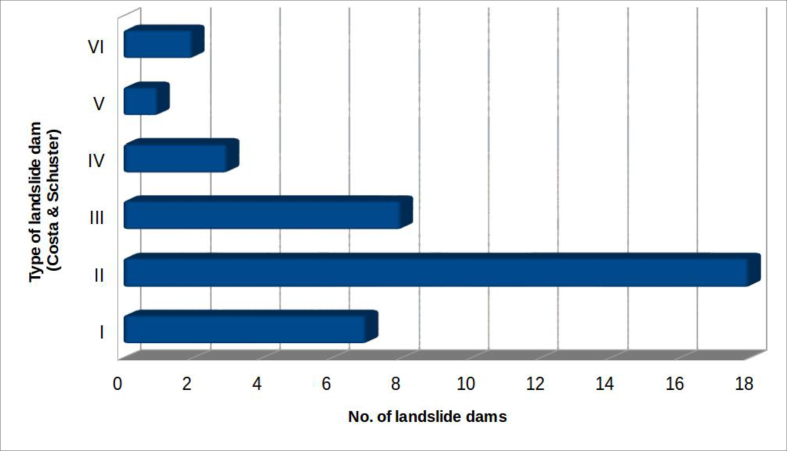
Distribution of landslide dams, based on the types derived from the classification by Costa and Schuster (1988).

Using the classification proposed by Costa and Schuster (1988), the landslides can be distinguished according to the relationship between the landslide accumulation (natural dam) and the affected valley floor. Total damming of the riverbed occurred in 39 cases out of the 53 surveyed (Figure 14).

Among these, type II are the most frequent (almost 46.2%), followed by type III (20.5%), type I (17.9%) and type IV (7.7%). Only two cases constitute type VI barriers: the first is the landslide at Bolognola, in the province of Macerata (Marche), which occurred in 1930 (Coppola et al., 1978; Dramis et al., 1988; Farabollini, 2010); the second is related to the landslide at Montelago, again in the Marche (province of Ancona) that deeply affected the entire valley floor, even going up the opposite side of the valley (Savelli et al., 2013). Finally, only one case among those surveyed belongs to the type V of Costa & Schuster: it is the landslide at Pian Martino, near Gubbio (province of Perugia), which originated as slump, and developed into multiple flows, generating two "cascade" lakes.

**Figure 15:**
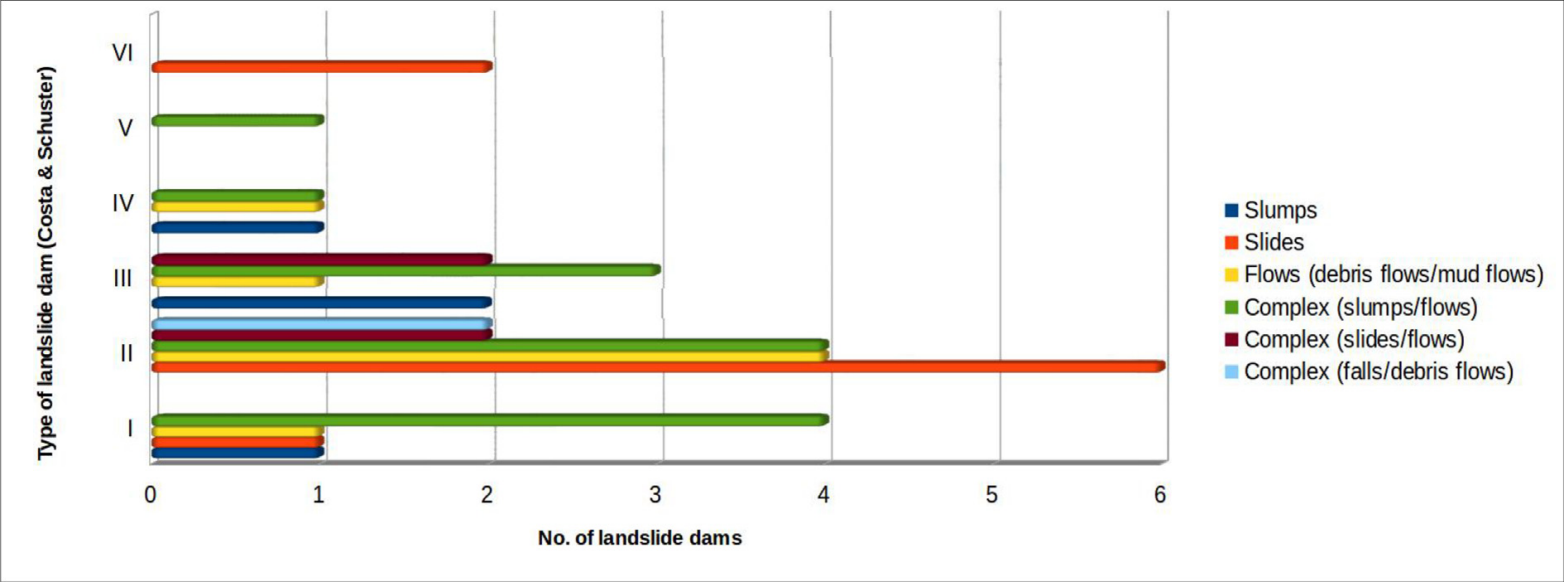
Relation between landslide types (WP/WLI, 1993) and landslide dam types, according to the Costa and Schuster (1988) classification.

Figure 15 highlights the relationship (in the 39 cases in which the barrier produced the formation of a lake) between the landslide types, as classified by Cruden & Varnes (WP/WLI, 1993) and the type of barriers classified according to Costa and Schuster (1988).

Restricting ourselves to the most numerous landslide types (I, II and III, 33 cases out of 39), it is noted that about 57% of those of the 1st type are due to complex slump/flow landslides. The landslides of the second type are roughly evenly distributed between slides (about 33.3%), flows (22.2%) and complex landslides of the slump/flow and slide/flow type (33.3%).

The type III cases are distributed between slumps, flows, and more frequently (62.5%) complex landslides (flows with slumps or slides).

#### 4.2.8 Formation of dam lake and its evolution

**Figure 16:**
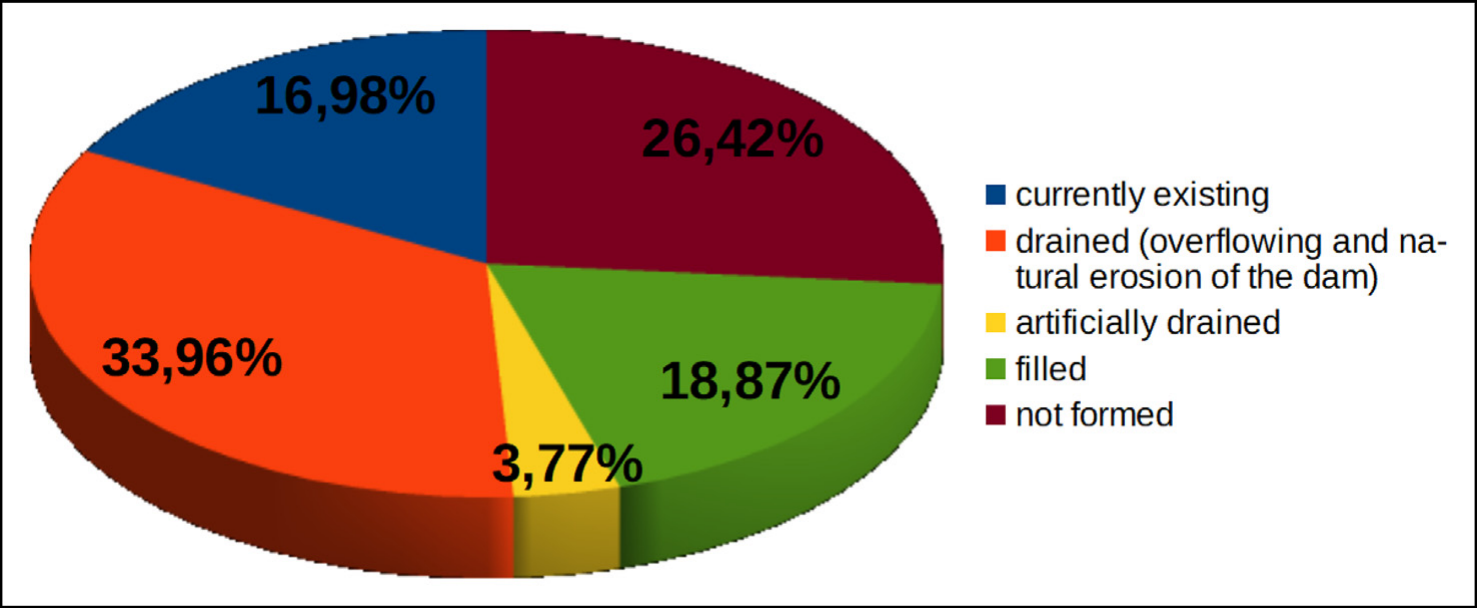
Evolution of the lakes eventually produced as results of the occlusions of the riverbeds, in % of the total landslides examined.

**Figure 17:**
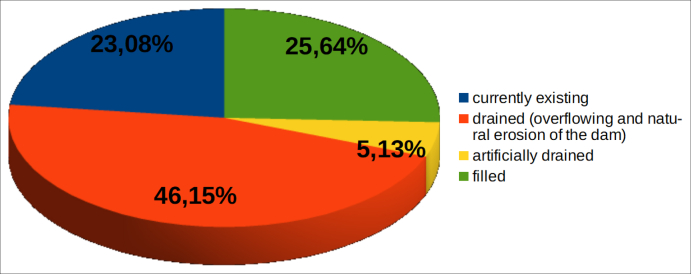
Evolution of the lakes eventually produced as results of the occlusions of the riverbeds, in % of the landslide dams.

In Figures 16 and 17 the evolution of the dam lake (if formed, due to the total damming of the river system) is represented.

Figure 16 shows that in 26.4% of the cases examined no lake formed. This occurred because:

- the stream bypassed the accumulation (there was a partial occlusion of the system);
- the material was quickly removed by the river flow;
- human intervention prevented the formation of a lake (2 cases).

In the remaining 73.6% of the cases (39 out of 53) the lake was formed but has undergone various evolutions. As shown in Figure 17:

− in about 46% of cases of total damming, the lake has emptied naturally, due to overflow and subsequent erosion of the occlusion barrier;
− in two cases (5%, Pieve S. Stefano and Valderchia), the lake was artificially emptied, as it posed a risk to downstream areas;
− in ten cases (25.6%) the dam itself persisted but the lake became completely filled by sediments and no longer exists;
− in nine cases (23%) the lake still exists. Three are in Umbria, on the Ventia Creek (landslide at C.se Sterpaiolo, province of Perugia), on the Burano River (landslide at Pian Martino, province of Perugia) and on the Cerreta Creek (landslide at Collepizzuto, province of Terni). Two are in Emilia-Romagna: the lake of Quarto, on the Savio River (artificially maintained by a dam built on the landslide body) and the lake produced by the landslide of Corniolo, on the Bidente River. The remaining 4 cases are in the Marche region: the landslide at Campo Lupo, which has occluded the Rio del Monte, in the basin of the Chienti River; the landslide at Porchiano, in the basin of the Tronto River; the two seismic-induced landslides (following the recent earthquake of 30 October 2016) at Visso, on the Nera River, and the Infernaccio Gorge, on the Tenna River.

We can conclude that almost half of the dam lakes have followed what is considered, in the literature, the most frequent evolution, i.e. the natural emptying due to overflow and progressive erosion of the natural barrier. Among other cases where the dam has persisted (19): about half have been completely filled, the remainder still exist.

## 5 Results: correlations between the analysed parameters

### 5.1 Evolution of the lake basing on the natural dam type

**Figure 18:**
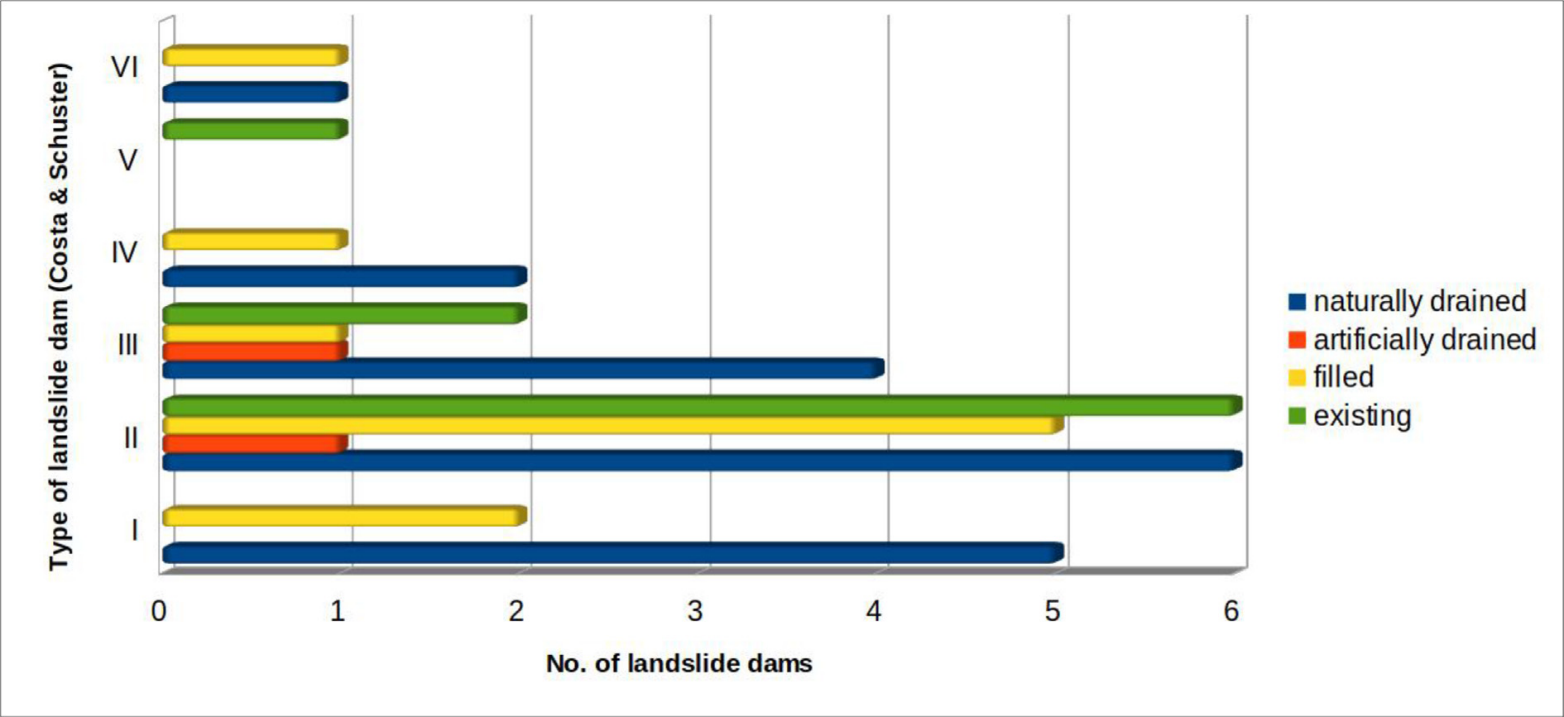
Correlation between types of landslide dams (according to Costa and Schuster, 1988) and evolution of the dam lake.

Figure 18 shows the 39 cases of total damming of the fluvial system that produced dam lakes. The chart relates the evolution of the lakes (drained naturally or artificially, filled or still existing) to the types of landslide dams as classified by Costa and Schuster (1988).

This figure shows that:

- lakes naturally drained by overflow and regressive erosion of the landslide body (the largest number - see Figure 20) are the result of landslides of types I, II, III or IV (only one case was of VI type);
- lakes that still exist are mostly are the result of landslides of types II or III (only one case was of V type).

### 5.2 Evolution of the phenomenon according to the width of the riverbed–alluvial plain system

**Figure 19:**
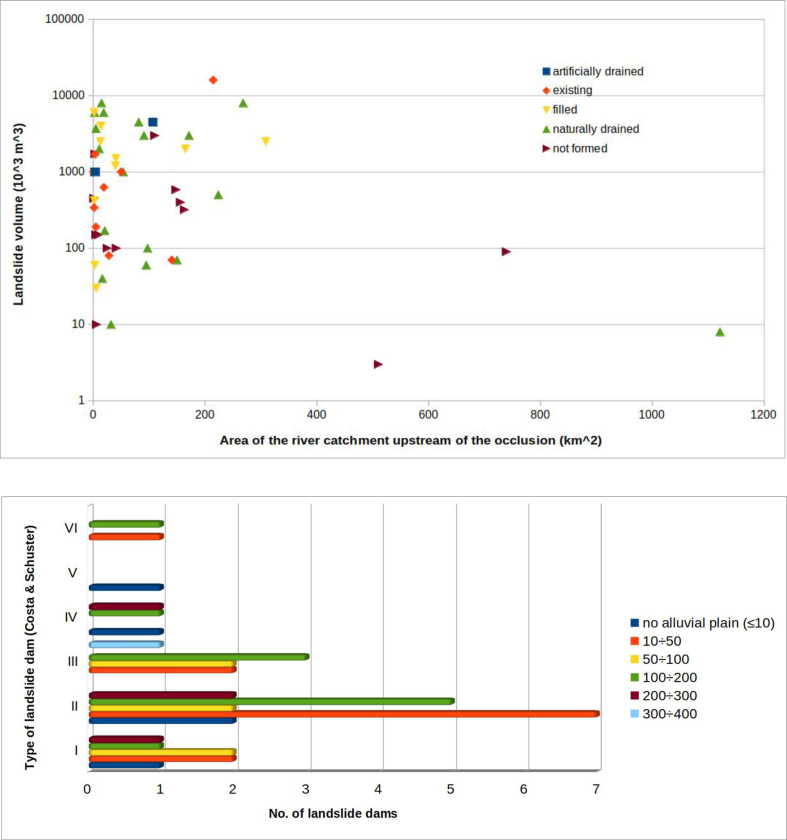
Correlation between types of landslide dams (according to Costa and Schuster, 1988) and width of the affected riverbed–alluvial plain system.

Figure 19 plots the 39 cases in which a dam lake occurred: the type of landslide dam is correlated with the classified width of the riverbed–alluvial plain system.

It can be seen that the most frequent cases (almost half, 18 out of 39) belong to type II and these mostly involve either fixed riverbeds (no alluvial plain) or relatively confined floodplains (less than 200 m wide).

### 5.3 Evolution of the phenomenon according to the volume of the landslide body and the area of the subtended river basin

**Figure 20:**
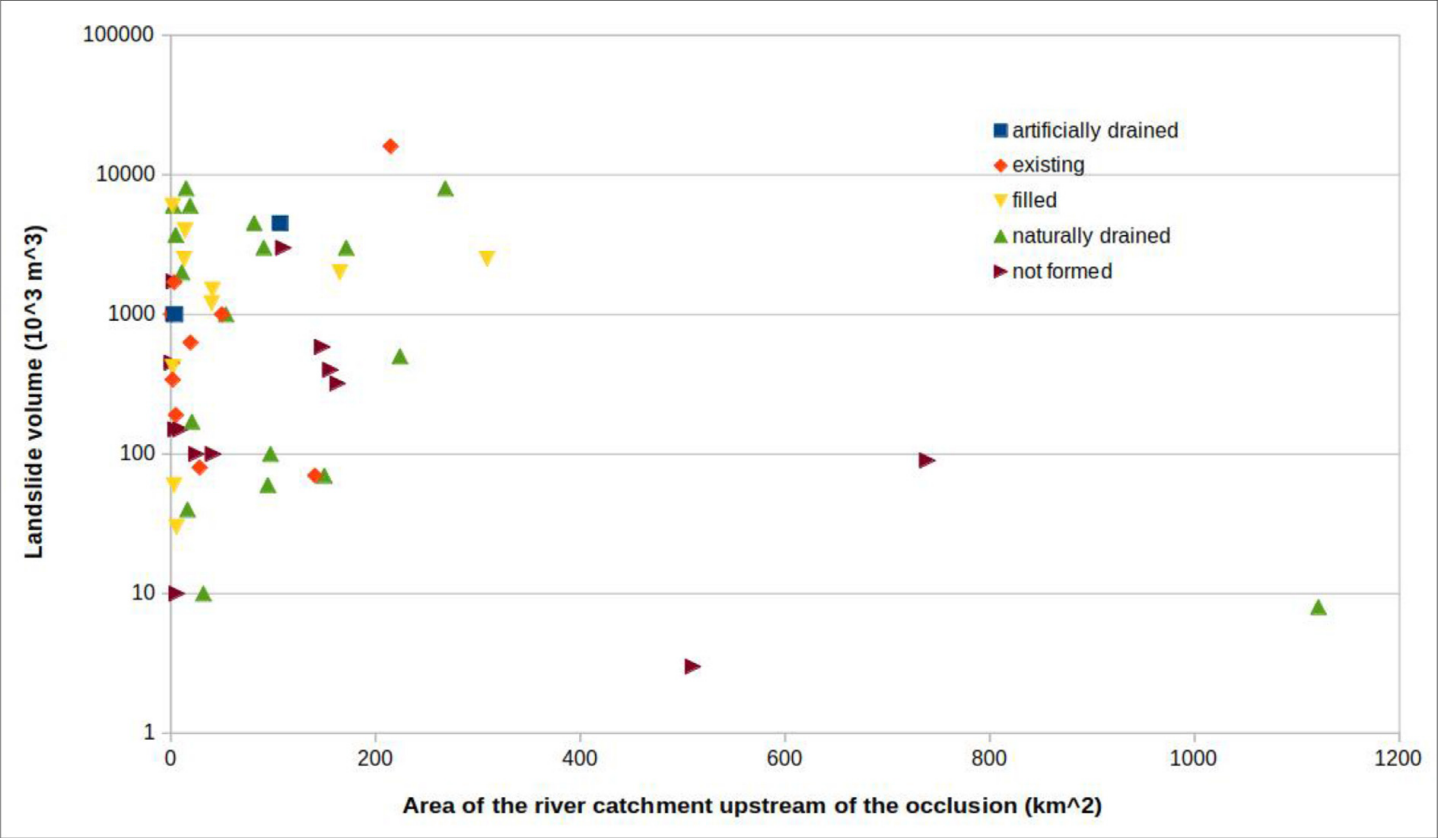
River catchment area upstream of the natural damvs volume of the landslide body. Basing on the available data, a field can be identified (to the left of the graph) which represents the most frequent combinations that can produce total occlusions of the riverbed, with the formation of a dam lake. Only one event (right at the bottom of the graph) “escapes” this rule. More scattered, however, is the combination of values of the two parameters which does not produce a dam lake.

The volume of the landslide body and the area of the river basin subtended by the dammed section are now compared. The data were inserted into a graph, specifying the formation or not of a dam lake and its evolution (Figure 20). The graph shows that the cases of “unformed lake” are characterized by quite dispersed combinations of volume of landslide and area of the basin subtended. In practice, it is not possible to estimate, with any reliability, under what circumstances a dam lake does not form.

**Figure 21:**
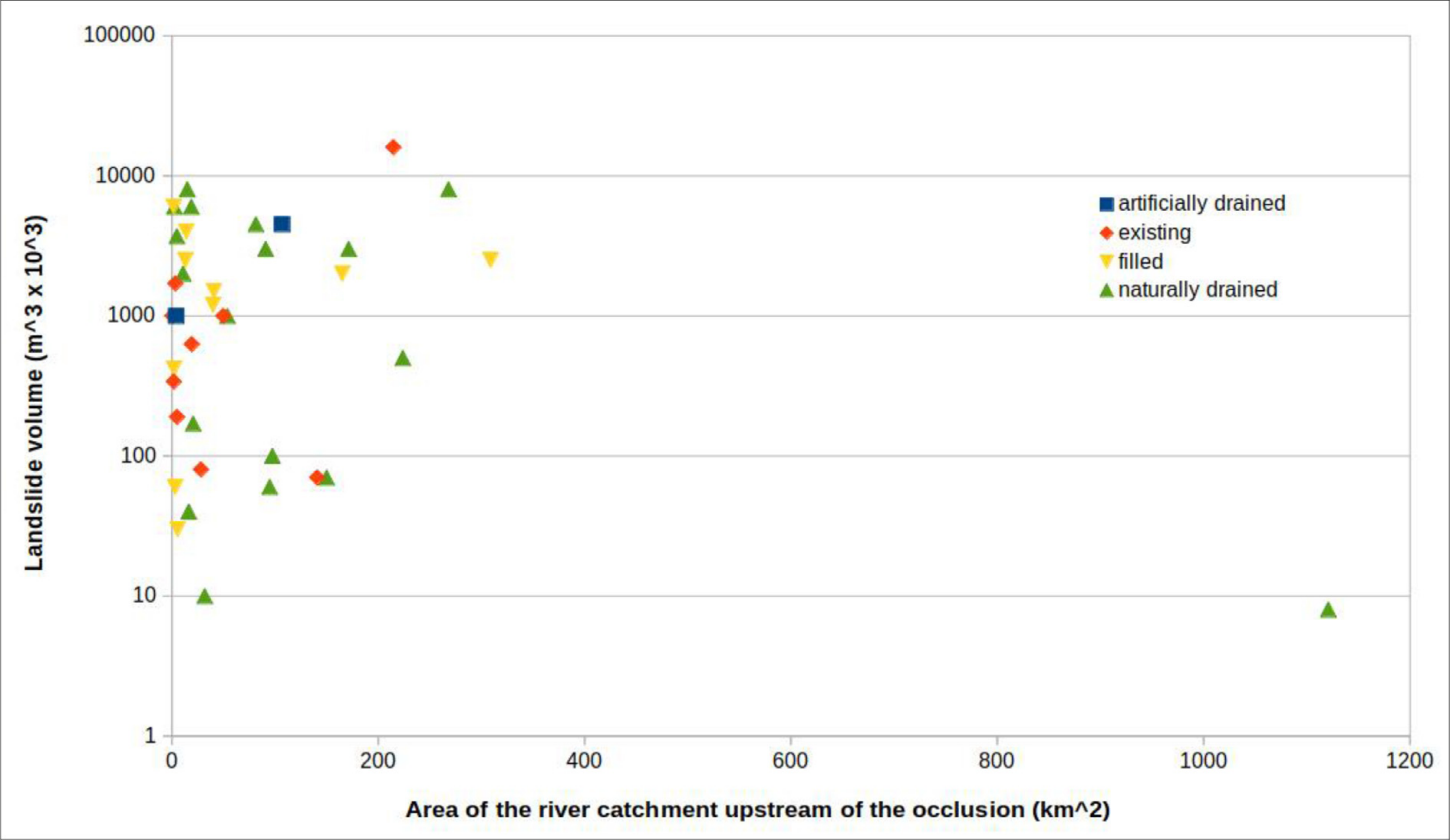
The same graph of Figure 23, without the cases of "unformed lake".

If the cases of “unformed lake” are excluded from the graph in Figure 20, the graph in Figure 21 is obtained. This indicates that the volume of the landslide body necessary to produce a total occlusion of the riverbed–alluvial plain system increases as the area of the river catchment upstream of the natural dam increases.

An explanation for this is that the area of the river basin is proportional to the flow discharge of the stream. Therefore, as relatively small landslide volumes can be easily removed by relatively high flows (which correspond to large basins), riverbed occlusion generally requires large volumes of material relative to the basin size.

In this way it is possible to distinguish in Figure 21 two fields: on the left, a field in which the combination of these values is favourable, even if not decisive (see discussion of the cases of “unformed lake” and Figure 20) for occlusion of the riverbed–alluvial plain system; on the right a field in which the combination of these values is not, in general, favourable for occlusion.

Only one case, among the 39 in which a dam lake was formed, does not follow this pattern. It had a particularly low ratio of landslide volume to catchment area upstream of the occlusion: it is the Piedipaterno landslide, in the lower right corner of Figure 21.

This exception can be explained, in the specific case, by both the type of landslide movement, extremely fast (it is a debris flow), and the narrowness of the section where the dam occurred (50 m - see Table 1).

### 5.4 Relationships between the landslide dam volume and the riverbed–alluvial plain width

**Figure 22:**
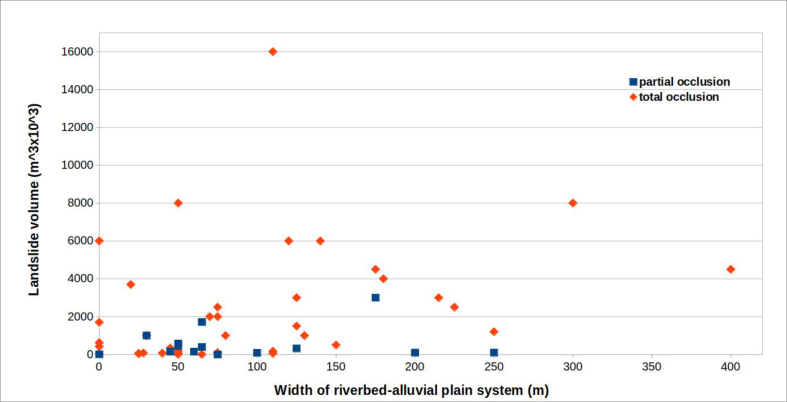
Relationship between the volume of landslides (distinguished as partial or total occlusion) and the width of the riverbed–alluvial plain system.

Figure 22 shows the relationship between the volume of landslide dams and the width of the affected riverbed–alluvial plain system.

The graph shows a dispersed relationship between the two parameters. In fact, especially for low values of both parameters, whether a dam lake will form (total occlusion) is not predictable. The likely explanation is that the velocity of the landslide is a significant factor in formation of a dam lake.

However, it is easy to identify a field in the chart, above volumes of about 3.0 million cubic metres, in which all the landslides produced a dam lake.

For lower volumes, in the bottom portion of the graph, the strong dispersion of points indicates that prediction of total occlusion, on the basis of these parameters, is impossible.

#### 5.4.1 Application of MOI index

Tacconi Stefanelli et al. (2016) proposed an index called MOI (Morphological Obstruction Index) with the aim of evaluation of the damming hazard caused by a landslide into a narrow valley. The MOI index is defined by (1):

(1) $$MOI=\text{log}\left(\frac{V_{l}}{W_{v}}\right)$$

where *V*_*l*_ represents the landslide volume (m^3^) and *W*_*v*_ the width of the valley (m).

Using this definition, the MOI index has been calculated for the whole dataset (53 landslides).

**Figure 23:**
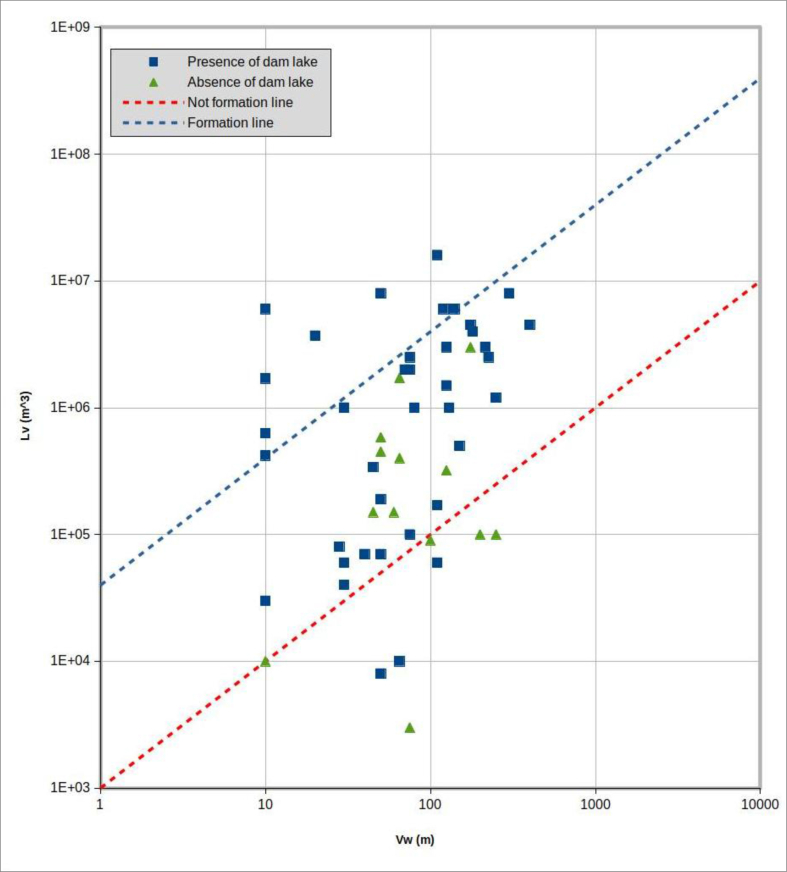
Plot of the entire dataset in XY log-log scatter plot. In X: Vw is the valley width and in Y: V_l_ is the volume of landslide in m^3^. The green triangles represent the events that did not produce a dam lake; the blue squares represent the events that produced a dam lake. The dashed blue line represents the “formation domain” limit, whereas the red dashed line represents the limit for the “non-formation” domain, as proposed by Tacconi Stefanelli et al. (2016).

Figure 23 shows the results obtained by log-log charting the case study values for *V*_*l*_ and *W*_*v*_. The lines on the chart delimit the fields in which, according to Tacconi Stefanelli et al. (2016), one should have either total occlusion, with the formation of a dam lake (upper left); partial occlusion, without dam lake formation (lower right); and an uncertainty band (central sector) in which it is not possible to make predictions, as there are cases of both total occlusion and partial occlusion.

Table 2 compares the MOI index predictions (i.e., the possibility total occlusion, with formation of a dam lake), with the outcomes of the cases reviewed here.

**Table 2:** Comparison of the outcomes in the whole landslide dataset with the MOI Index predictions.

MOI index	Presence of dam lake		Total
	No	Yes	
Formation domain (MOI >4.6)		9	**9**
Non formation (MOI <3)	4	3	**7**
Uncertain (3 < MOI <4.6)	10	27	**37**
**Total**	14	39	**53**

Out of a total of 53 landslides, 37 of them (70%) fall into the MOI index uncertainty area. In the MOI index formation domain the forecast on 9 real cases is always correct. The MOI index non-formation area is not so accurate: among the 7 landslides predicted to not form a dam, 3 did in fact produce a dam lake.

The MOI index is generally not very reliable, in its defined purpose, at least for the dataset considered here.

## 6 Discussion and conclusions

The census of landslide dams and creation of a database is an important step in any methodology for the analysis of the related hazard. The process can be applied, in the future, to other cases that may be already verified, or to current (or future) cases for which there not yet a known outcome.

The census showed that:

- Most of the landslides that produced total occlusion, with the formation of a dam lake (almost 85%) involved less than 5 million m^3^ of material, while a third of total occlusions were produced by landslides with a volume between 1 and 5 million m^3^.
- The prevailing landslide types are slides/slumps and flows, or some combination of the these (complex or composite landslides).
- Occlusions of types I, II and III, of the classification by Costa and Schuster (1988), are clearly prevalent.
- The geological formations most affected are in facies of siliciclastic flysch (alternation of sandstones and marls). The reason why these formations are affected more than others is that siliciclastic formations already have a high index of landslide susceptibility. In fact, the erosive processes of the streams in the area create mainly vertical erosion and this, in the particularly erodible (marly and arenaceous) lithotypes, has led to entrenchment of the riverbeds and high relief energy, which are predisposing factors for landslides. This is supported by the high percentage of episodes of this kind that involved riverbed–alluvial plain systems in narrow valleys (less than 50 m) with entrenched riverbeds. Another predisposing factor is the behaviour of the aforementioned materials during the landslide movement: rocks of the siliciclastic flysch, once they reach the valley floor, tend to expand, as the clay-rich materials strongly absorb water, producing landslide bodies larger than those of similar movements set, for example, on limestone. The latter, generally, are characterized by slides and falls of large blocks, and form more circumscribed landslide bodies. A third cause is the lower permeability of landslide body materials, in siliciclastic flysch, relative to other types of rocks such as limestones which have only a minor clay fraction. The difference is due to the size of the constituent materials: an accumulation of marly and arenaceous rocks will have a lower permeability than that of calcareous material. In the latter, the greater competence of the lithotype produces a complex of larger stone elements allowing greater passage of fluids (high permeability).
- The relatively low frequency of landslide dams in the Plio-Pleistocene continental sediments, in fluvio-lacustrine facies, can be attributed, firstly, to the smaller extent of soils of this type relative to flyschoid formations, and secondly to the type of material, which is often very fine and therefore easily re-mobilizable.
- The most important landslide triggers are climatic-meteorological (heavy precipitation, rapid melting of snow) and geological-structural (unfavourable orientation of the layers, fractured material, contrast of competence between lithotypes).
- In only two cases were there victims: Pieve S. Stefano (1855) and Quarto di Savio (1812). In the first case four people drowned because they were surprised by the rapid rise of the lake level and were trapped in their homes; in the second, the landslide directly overwhelmed and killed eighteen people. As regards damage to property, the landslide of S. Agata Feltria was the most catastrophic event: the landslide crossed the inhabited centre dividing it into two parts. Other significant damage occurred in S. Piero in Bagno and in the aforementioned Pieve S. Stefano (1855), where the houses were submerged by the rising lake. In four cases human interventions sought to empty the lake or prevent its formation. In the case of Quarto di Savio, on the other hand, an artificial dam was built over the natural dam for hydroelectric purposes.
- Almost half of the produced lakes has the same common evolution, as also reported in the literature, i.e. natural emptying due to overflowing and progressive erosion of the natural threshold. When the barrier resisted, complete filling of the lake with sediments occurred in about half of the cases; in the remaining cases, the lake still exists.

Some important factors affecting the occurrence of landslide dams (and their hazard) were tested for correlation. The results provide useful provisional indicators.

The most interesting of these correlations, based on the evolution of the lake and the type of landslide, are those that compare the volume of landslide with the area of the catchment upstream of the occlusion or with the width of the riverbed–alluvial plain system. The graphs show important points for consideration: almost all cases of occlusion are located in a well-defined area of the graph in Figure 23. This indicates that, in order to have occlusion, the volume of the accumulation must increase as either the width of the system or the area of the river catchment upstream of the occlusion increase. This means that, given a certain volume of landslide material, there is generally the possibility of occlusion when the area of the basin (and, therefore, the flow discharge of the stream) is below a certain threshold; beyond this threshold occlusion occurs very rarely. Under the same relationship, given a certain section of riverbed that subtends a basin area, until the landslide reaches a certain accumulation volume, full occlusion of the riverbed is very unlikely. In practice, the graph permits important conclusions about the predictability of lake formation. The relationship between the volume of the landslide and the area of the basin subtended by the barrier, as shown in Figure 21, allow identification of "trigger thresholds".

However, one case, among those examined in Figure 20, escaped this general rule: it is the debris flow of Piedipaterno, on the F. Nera (Conversini et al., 2005), where a landslide with small volume made an occlusion even though the subtended basin was relatively large.

This is evidence of how, in some cases, other elements, in addition to the parameters considered, can come into play. These include the landslide velocity and the width of the riverbed–alluvial plain system at the barrier location. Figure 21 shows the same data as Figure 20, excluding the cases of "unformed lake". This figure, which plots the parameters landslide volume and area of the river catchment upstream of the occlusion, confirms the prior differentiation in two distinct fields. In the upper left corner the conditions that favour the formation of a landslide dam, and in the lower right corner the combinations for which the formation of a dam lake is unlikely, except in the case of extremely fast landslides (i.e., flows).

The predictability of full occlusion is more difficult using the parameters “volume of the landslide” and “width of the riverbed–alluvial plain system”: here too landslide velocity may be decisive (Figure 22).

It can therefore be concluded that:

1) a high ratio of landslide volume to the river catchment area upstream of the occlusion is a predisposing condition for the occurrence of total occlusion of rivers by landslides, but not sufficient. Cases of “unformed lake” (partial occlusions) can also occur in conditions with this high ratio (see Figure 20). These exceptions were commonly slumps and slides, not fast landslides, and the stream, evidently, had sufficient energy to immediately dispose of the material that had invaded the riverbed, preventing formation of a dam lake;
2) the velocity at which the landslide moves is one of the most decisive factors in occlusion of the riverbed. Figure 21, with a hypothetical line dividing two distinct fields, is valid only for landslides that are not extremely fast. Indeed, rapid debris flows, even with relatively small volumes, can cause total obstruction, regardless of the river flow discharge, especially when the river cross-section is small.

It would be ideal to be able to define indexes that can, on the basis of selected parameters, be used to define the landslide dam hazard in a quantitative manner. We tested the MOI index, defined by Tacconi Stefanelli et al. (2016), which appears promising because it is based on evaluation of two parameters that seem to have potential for successful prediction of landslide dam hazard: the volume of the landslide and the width of the riverbed system–alluvial plain affected by the landslide (Figure 23).

However, comparison between MOI forecasts and the dataset used here demonstrated that this index is insufficient to determine, unequivocally, whether total occlusion of the riverbed will occur. Indeed, about 70% of the cases studied fall within the “uncertainty band” of the MOI, not allowing any forecast. Among the remaining 30%, only 9 cases of total riverbed occlusion (17% of the total dataset) did the MOI provides reliable forecasts. Predictions of “non-formation” of a dam lake had even less certainty, in 3 out of 7 cases, in which no dam would be predicted by MOI, a dam lake occurred.

MOI thus seems to make reliable forecasts only in cases above the formation line, i.e., when the values of the two parameters (landslide volume and width of the river system) gives an MOI >4.6. For MOI values < 4.6, such a high degree of uncertainty remains, as to render the index itself unusable.

The lack of applicability of MOI, at least for the cases considered here, demonstrates the difficulty of defining generic indexes. While valid in intent, these can be revealed, if applied to specific territorial realities, to be completely or largely unreliable.

Determination of a generic index is made problematic by the peculiar characteristics of each territorial area, from lithological, geomorphological and geological-structural point of views. The extreme geological variability that characterizes the Italian peninsula makes it unsurprising that the MOI, proposed as valid for all Italy, has been found inapplicable in the central-northern Apennine area.

It is our opinion that such indexes, when they do not lose their intrinsic reliability (as in this case), must at least be identified with, and applied to, specific and restricted territories: they cannot be generalized.

## Declarations

### Author contribution statement

C. Cencetti: Conceived and designed the analysis; Analyzed and interpreted the data; Contributed analysis tools or data; Wrote the paper.

P. De Rosa: Analyzed and interpreted the data; Wrote the paper.

A. Fredduzzi: Performed the experiments; Analyzed and interpreted the data; Contributed analysis tools or data; Wrote the paper.

### Funding statement

This work was supported by Regional Administration of “Regione Umbria” (3164/11).

### Competing interest statement

The authors declare no conflict of interest.

### Additional information

No additional information is available for this paper.
